# Interventions for the prevention and management of occupational stress injury in first responders: a rapid overview of reviews

**DOI:** 10.1186/s13643-020-01367-w

**Published:** 2020-05-31

**Authors:** Jesmin Antony, Raman Brar, Paul A. Khan, Marco Ghassemi, Vera Nincic, Jane P. Sharpe, Sharon E. Straus, Andrea C. Tricco

**Affiliations:** 1 Knowledge Translation Program, Li Ka Shing Knowledge Institute, St. Michael’s Hospital, 209 Victoria Street, East Building, Toronto, Ontario M5B 1 W8 Canada; 2 Department of Geriatric Medicine, University of Toronto, Toronto, Ontario Canada; 3 Epidemiology Division, Dalla Lana School of Public Health and Institute for Health, Management, and Evaluation, University of Toronto, Toronto, Ontario Canada

**Keywords:** First responders, Interventions, Occupational stress injury, Police, Firefighters

## Abstract

**Background:**

First responders are a high-risk population for occupational stress injuries as they often encounter prolonged stress within their line of work. The aim of this rapid overview of reviews is to summarize existing evidence on interventions for the prevention and management of occupational stress injury (OSI) in first responders.

**Methods:**

MEDLINE, EMBASE, PsycINFO, CINAHL, Web of Science, and Cochrane Library were searched for systematic reviews examining the impact of prevention, rehabilitation, and resilience-building strategies targeting frontline community safety personnel in February 2019. Pairs of reviewers screened titles and abstracts followed by full-text articles and conducted data abstraction and quality appraisal using the AMSTAR II tool. To ensure a rapid overview process, the search strategy was limited to the last 10 years, quality appraisal of reviews and abstraction of study-level data was completed by one person and verified by another, and the quality of the individual primary studies was not appraised. The findings were summarized descriptively.

**Results:**

A total of 14 reviews with 47 unique primary studies were found after screening 1393 records. A majority of studies targeted OSI in police officers (78.7%), followed by firefighters (17%) and correctional officers (4.3%). Of the 47 included primary studies, 24 targeted prevention of OSI (i.e., resilience training, stress management, suicide prevention, and other health promotions) and 23 targeted rehabilitation (i.e., drug therapy, psychotherapy, and other therapies). Prevention strategies including resilience training programs had positive outcomes, while suicide prevention and psychotherapy interventions reported mixed results.

**Conclusions:**

Some promising interventions targeting the prevention and rehabilitation of OSI among police officers, firefighters, and correctional officers were identified in the included studies, and these results will serve as a basis for the development of evidence-based strategies to mitigate future risks in this population. However, several gaps were also identified in this area that will require further investigation prior to widespread implementation of effective interventions.

**Systematic review registration:**

PROSPERO CRD42019125945

## Background

Occupational (or operational) stress injury (OSI) describes a broad range of psychological and other conditions resulting from duties performed on the job that interferes with a person’s professional and personal life, including anxiety, depression, and post-traumatic stress disorder (PTSD) [1, 2]. First responders are a particularly susceptible population to these injuries, as they often encounter high-risk situations and must deal with daily, routine stressors within their line of work [3]. Although there is a large amount of literature on OSI among first responders, research has traditionally reported on military personnel, due to the extremely high rates of PTSD and associated conditions they experienced in the late twentieth century and early 2000s [4–6]. More recently, research focus has shifted to include paramedics and emergency medical services (EMS), noting that this population has similar patterns of stress injury as those seen in veterans [7, 8]. However, there has been less focus on groups categorized as frontline community safety personnel, such as police officers and firefighters, and especially correctional officers and coroners.

The impact and cost of OSI are well-documented. There are substantial direct and systemic costs, such as cost to individuals affected and their primary caregivers, cost of healthcare providers for treatment of OSI-related ailments, and the cost of lost labor and productivity of officers taking medical or stress leave [3]. This project was commissioned by the Ontario Ministry of Community Safety and Correctional Services (MCSCS), to assess the utility of interventions currently implemented at the workplace targeting OSI as a first step to the development of evidence-based action plans to mitigate future risks in this population.

To address this need for evidence, we conducted a rapid overview of reviews (hereafter called overview) summarizing the usefulness of existing strategies for the prevention and management of OSI targeting frontline public/community safety personnel.

## Methods

An overview (i.e., a synthesis of systematic review findings) is an effective method to systematically gather, appraise, and summarize existing evidence on a broad topic that has been well-studied, and identify gaps in the research efforts to date [9]. This overview includes systematic reviews targeting first responders or frontline community safety personnel, including police officers, firefighters, correctional officers, and coroners, with a focus on prevention and rehabilitation of OSI.

To ensure methodological rigor, the review team followed guidelines outlined by the Cochrane Handbook chapter regarding Overview of Reviews [10, 11] and applied the Preferred Reporting Items for Overviews of Systematic Reviews Including Harms (PRIO-harms) checklist (Additional File 1) for transparency of our methods [12]. However, to meet the rapid, 10-week timeline needs of our primary knowledge user (MCSCS), some streamlined steps were taken. Specifically, the search strategy was limited to reviews in English published in the past 10 years (2009 onwards), and rather than appraising the methodological quality of each review in duplicate, one person appraised the included reviews and another verified the appraisal for accuracy. As such, the methodology was considered to be a rapid (or streamlined) overview.

### Protocol registration

A protocol for the overview was developed *a priori* and registered on the PROSPERO database (registration no. CRD42019125945). However, upon starting the review, a few minor modifications were made to the outlined methods. For example, we added “rapid” to the title of the overview of reviews for transparency of methods, and we chose to define first responders using inclusive terminology relevant to the MCSCS (i.e., frontline public/community safety personnel).

### Eligibility criteria

Study eligibility criteria developed using the PICOS (population, intervention, comparator, outcome, and study) approach [13] were as follows:

#### Population

Populations of interest included frontline community safety personnel (i.e., police and firefighters), coroners/forensic pathologists, and correctional service employees. As it was not of relevance to our knowledge user, reviews that only included EMS personnel were not included. However, reviews including a mix of frontline community safety personnel were included.

#### Interventions

Interventions of interest were prevention strategies (e.g., training and learning approaches, standards of practice, surveillance of risk factors, self-assessments, screening protocols), rehabilitation (e.g., therapeutic interventions, digital interventions, pharmaceutical interventions, psychological interventions, organizational support systems, peer support programs), and resilience-building strategies (e.g., shifting organizational culture, mental health promotion, overcoming organizational barriers, leadership/management training, overcoming stigma) targeting OSI.

#### Comparators

Any comparisons to the interventions listed above, including usual care or no treatment, were eligible for inclusion.

#### Outcomes

Primary outcomes of interest were the effects of the interventions on mental health status, including but not limited to the following: OSI, trauma, anxiety, depression, mood, addiction/substance abuse, PTSD, stigma, suicidal ideation/behavior, time lost from work, and physiological responses to trauma such as increased pulse/heart rate or fatigue/sleepiness. Healthy and maladaptive habits and general health and job satisfaction outcomes were also considered.

#### Study design(s)

Included studies were systematic reviews, defined by the Cochrane Handbook as a systematic synthesis of empirical evidence [11] of interventions.

#### Others

Our literature search was limited to reviews published in English in the last 10 years; however, individual studies included in these reviews were not restricted by publication date.

### Study selection

Our search strategy was developed by an information specialist and peer-reviewed by another using the Peer Review of Electronic Search Strategies (PRESS) checklist [14]. MEDLINE, EMBASE, PsycINFO, CINAHL, Web of Science, and Cochrane Library databases were searched on February 17, 2019, for relevant reviews. The search was limited to include English-language reviews published in the past 10 years (i.e., 2009 onwards). The search strategy for MEDLINE can be found in Appendix B (Additional File 1). To further ensure that all relevant reviews were included, the reference lists of included reviews were scanned for additional citations.

Titles and abstracts (level one) and full-texts (level two) were screened for relevance by two reviewers independently using the synthesi.SR tool [15] developed by the Knowledge Translation Program, St. Michael’s Hospital of Unity Health Toronto. To ensure consistency among reviewers, one pilot test was conducted prior to both levels of screening. This entailed screening of 100 citations by the team at level one with 86% agreement and screening of 20 full-text articles at level two resulting in 75% agreement. Subsequently, pairs of reviewers proceeded to screen the remaining articles independently at each level of screening. Discrepancies were resolved through discussion or by a third reviewer when necessary.

### Data abstraction

A standardized data abstraction form was developed based on predefined eligibility criteria. Data from each review were abstracted on characteristics (e.g., year of conduct, number of included studies, type of included study designs, sample size), interventions examined (e.g., type of intervention, duration, frequency), and outcomes examined (e.g., name of outcome, outcome measure/definition). The team piloted the data abstraction form on five articles. After meeting and discussing discrepancies, pairs of reviewers independently abstracted data from the included articles. Discrepancies were resolved by a third reviewer.

In order to supplement the review-level data and provide the commissioning agency with more information on the interventions and outcomes examined, the review team took an additional step of abstracting relevant data from each of the primary studies, including specific components of the interventions and effect sizes and significance levels of the outcomes examined. These data were abstracted by one reviewer and verified by a second reviewer. A table was also created to determine the extent of overlap in primary studies included across the reviews.

### Quality appraisal

AMSTAR 2 (A Measurement Tool to Assess Systematic Reviews version 2) [16] is a 16-item critical appraisal tool that we utilized to assess the methodological quality of all included reviews. An experienced reviewer independently read and appraised the risk of bias for each review, while a second reviewer verified the results. Discrepancies were resolved by discussion or by a third reviewer, if needed. Response options for the AMSTAR 2 included “yes,” “partial yes,” and “no.” In order to reply “yes” or “partial yes,” the review had to meet all of the criteria specified by the tool. A “no” was used to indicate that the criteria for either “yes” or “partial yes” were not met or there was an absence of the item overall.

An overall score was also given to each review to indicate whether the review was of high, moderate, low, or critically low quality. The overall score was based on the number of “no” responses to the pre-defined critical checklist items (items 2, 4, 7, 9, 11, 13, 15), indicating “critical flaws” within the review’s design or conduct. The maximum score a review could receive was “high” quality if there were no critical flaws, then “moderate” quality if there was one critical flaw, followed by “low” quality if there were two critical flaws, and the lowest possible score on the tool was “critically low” quality if there were more than two critical flaws.

### Data synthesis

The results of the included reviews and primary studies are summarized descriptively. The data are presented in tables to allow for comparisons across populations, interventions, and outcomes in the following results section.

## Results

### Literature search

**Fig. 1:**
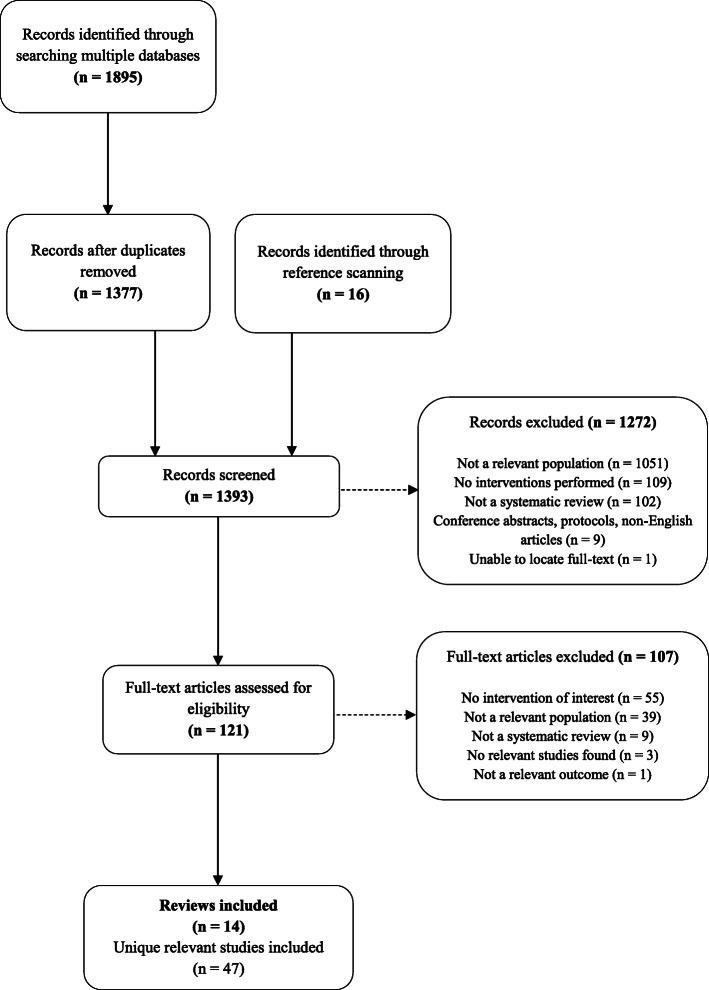
Rapid overview of reviews flow chart

A comprehensive database search identified 1377 records, with an additional 16 located through scanning the reference lists of the included reviews. In total, 1393 titles and abstracts were screened for eligibility at level one and 121 full-text articles at level two. Fourteen relevant reviews (including 47 unique primary studies) met eligibility criteria and data were abstracted from these articles. The full study flow, with reasons for exclusion, is provided in Fig. 1.

### Review characteristics

The 14 included reviews were conducted from 2009 to 2019 and the corresponding authors were predominantly located in Australia (42.9%) and the United States (USA) (28.6%), followed by Canada (14.3%), the Netherlands (7.1%), and the United Kingdom (UK) (7.1%) (Table 1). Of the 14 included reviews, only two included a single population group of police officers, the other 12 included mixed populations (e.g., military workers, nurses, volunteers).

**Table 1:** Review and primary study characteristics

Characteristics	*N* (%)
**No. of included reviews**	**14**
**Date of publication**	
2009–2012	4 (28.6%)
2013–2016	5 (35.7%)
2017–2019	5 (35.7%)
**Country of publication**	
Australia	6 (42.9%)
Canada	2 (14.3%)
Netherlands	1 (7.1%)
UK	1 (7.1%)
USA	4 (28.6%)
**No. of included primary studies**	**47**
**Study design**	
Randomized controlled trials	19 (40.4%)
Non-randomized controlled trials	6 (12.8%)
Quasi-experimental	7 (14.9%)
Observational	9 (19.2%)
Case report	5 (10.6%)
Descriptive	1 (2.1%)
**Population**	
Police	37 (78.7%)
Firefighters	8 (17.0%)
Correctional services	2 (4.3%)
Coroner/forensic pathologists	0 (0.0%)
**Intervention**	
Prevention	24 (51.1%)
Rehabilitation	23 (48.9%)

Within the 14 reviews, we identified 47 unique primary studies, examining both a relevant first responder population and an intervention targeting OSI. The majority of the studies focused on police (78.7%) and firefighters (17%) with only a small percentage focusing on correctional services (4.3%). No relevant coroner/forensic pathologist studies were found and none of the included studies reported multiple relevant populations. In total, 11 of the 47 included studies overlapped, 10 of which were found in two of the included reviews, and 1 was found in 4 of the included reviews as presented in Appendix C (Additional File 1). The majority of included primary studies were RCTs (40.4%), followed by observational (19.2%), quasi-experimental (14.9%), NRCT (12.8%), case reports (10.6%), and a descriptive study (2.1%). All were conducted between 1979 and 2017.

### Summary of included reviews

Table 2 summarizes the 14 included reviews. Although many of these reviews included a large number of primary studies, only a small subset was directly relevant to this overview. The excluded primary studies (Additional File 1) either included populations that were not relevant to our overview (e.g., non-first responder populations) or did not include interventions targeting OSI (e.g., study on the prevalence of PTSD). The citations of the 47 relevant primary studies included in our overview are listed in Table 2 and further details on each study can be found below.

**Table 2:** Review summaries

Review author (year)	Review country	Review objective(s)	Total no. of studies included in review (no. of relevant studies)	Citations of relevant studies (author, year)	Population(s) included in relevant studies	AMSTAR 2 score
Lees, T (2019) [17]	Australia	To review the most recent information regarding anxiety, PTSD, sleepiness, and fatigue and to identify interventions and treatments proposed to overcome work-related stressors and associated mental illnesses inflicting law enforcement officers.	43 (6)	Arble, 2017; Chongruksa, 2012; Christopher, 2016; Oliver, 2009; Peres, 2011; Plat, 2013	Police	Critically low
Barger, LK (2018) [18]	USA	To critically review and synthesize existing literature on the impact of fatigue training on fatigue-related outcomes for emergency medical services (EMS) personnel and similar shift worker groups.	18 (4)	Christopher, 2016; Holbrook, 1994; Kuehl, 2016; Sullivan, 2016	Police, firefighter	Moderate
Torchalla, I (2018) [19]	Canada	To summarize the evidence base for interventions targeting individuals with work-related post-traumatic stress disorder (PTSD), to make recommendations for clinicians and administrative decision-makers involved in their rehabilitation, and to guide future research in this area. Particular attention was given to studies that were conducted in naturalistic clinical settings or in a workers’ compensation claim context.	11 (2)	Gersons, 2000; Lansing, 2005	Police	Critically low
MacMillan, F (2017) [20]	Australia	To systematically review studies of health promotion interventions in the police force.	21 (7)	Jeter, 2013; Kuehl, 2016; Norris, 1990; Norvell, 1993; Richmond, 1999; Short, 1984; Tanigoshi, 2008	Police	Low
Witt, K (2017) [21]	Australia	To summarize the international literature on the effectiveness of suicide prevention programs for protective and emergency services employees.	13 (4)	Finney, 2015; Levenson, 2010; Mishara, 2012; Welch, 1998	Police, firefighter	Critically low
Rose, FR (2016) [22]	USA	To conduct a systematic review and meta-analysis with regards to the effectiveness of psychological debriefing.	27 (8)	Bohl, 1991; Bohl, 1995; Carlier, 2000; Harris, 2002; Leonard, 1999; Regehr, 2001; Ruck, 2013; Tuckey, 2014	Police, firefighter, correctional services	Critically low
Whybrow, D (2015) [23]	UK	To summarize current knowledge about TRiM and make recommendations for further research.	13 (2)	Hunt, 2013; Watson, 2014	Police	Critically low
Milner, A (2014) [24]	Australia	To provide a systematic assessment of workplace suicide prevention activities, including short-term training activities, as well as suicide prevention strategies designed for occupational groups at risk of suicide.	13 (1)	Mishara, 2012	Police	Critically low
Patterson, GT (2014) [25]	USA	To conduct a systematic review examining the effects of stress management interventions on outcomes among police officers and recruits.	12 (12)	Ackerly, 1986; Coulson, 1987; Digliani, 1994; Gersons, 2000; Ireland, 2007; McCraty, 1999; Norvell, 1993; Richmond, 1999; Shipley, 2002; Short, 1984; Tanigoshi, 2008; Wilson, 2001	Police	Critically low
Skeffington, PM (2013) [26]	Australia	To conduct a systematic review in order to identify and synthesize all programs aimed at the primary prevention of PTSD to date.	7 (3)	Arnetz, 2009; Sarason, 1979; Sijaric-Voloder, 2008	Police	Critically low
Haugen, PT (2012) [27]	USA	To conduct a systematic review of the PTSD treatment literature (English and non-English) in order to evaluate such treatment proposals based on what is known about treating PTSD in first responders. This review especially sought to identify RCTs whose primary outcome was PTSD.	17 (8)	Cornelius, 2007; Coupland, 2009; Ford, 1996; Gersons, 2000; Kitchiner, 2004; Lansing, 2005; Tolin, 1999; Wilson, 2001	Police, firefighter	Critically low
Plat, MJ (2011) [28]	Netherlands	To conduct a systematic review describing (1) the existing job-specific workers’ health surveillance (WHS) activities, and (2) the effectiveness of job-specific WHS interventions with respect to work functioning, for selected jobs.	31 (2)	Arnetz, 2009; Elliot, 2007	Police, firefighter	Low
Stergiopoulos, E (2011) [29]	Canada	To identify interventions targeting work-related PTSD in order to get workers back to the workplace.	7 (1)	Gersons, 2000	Police	Moderate
Martin, A (2009) [30]	Australia	To investigate whether different types of health promotion intervention in the workplace reduce depression and anxiety symptoms.	22 (1)	McCraty, 2003	Correctional services	Critically low

*EMS* emergency medical services, *PTSD* post traumatic stress disorder, *RCT* randomised controlled trial, *TRiM* trauma risk management

### Quality appraisal of included reviews

A summary of the quality appraisal results of the 14 included reviews using the AMSTAR 2 tool can also be found in Table 2. A more detailed quality appraisal table can be found in Appendix D (Additional File 1), which includes the responses for each of the 16 items on the AMSTAR 2 tool. Two of the reviews could not be appraised for quality because they did not find studies reporting results of an intervention. The majority of the reviews were rated critically low (71.4%) or low (14.3%) in terms of the overall review quality. Two reviews were rated moderate (14.3%) quality. The most common critical flaws within the low-quality reviews were the lack of a protocol or an a priori design (item 2) and no list of excluded studies (item 7). A large portion of the low-quality reviews did not complete a risk of bias assessment for their included studies (item 9), and therefore, risk of bias was also not considered when interpreting the results (item 13).

### Data synthesis

Since the included systematic reviews provided few details on the interventions and outcomes assessed in their primary studies, we abstracted this information from the relevant primary studies themselves. In the following section, the 47 unique primary studies reporting any intervention are organized by study population.

#### Police

Twelve reviews included 37 unique studies examining interventions targeting OSI in police officers. Summaries of the included police primary studies are presented in Appendix E1 (Additional File 1), including the studies’ objectives, methods, results, and conclusions.

#### Firefighters

Interventions for firefighters were examined in six reviews, including eight primary studies, and summarized in Appendix E2 (Additional File 1).

#### Correctional services

The two intervention studies for correctional officers we found in two reviews are summarized in Appendix E3 (Additional File 1).

### Interventions and outcomes examined

In this section, the 47 primary studies are categorized by intervention type and a summary of each study, including relevant outcomes, is provided below. A description of each of the study populations, interventions, and findings are also presented in Table 3 (prevention interventions) and Table 4 (rehabilitation interventions).

**Table 3:** Interventions for prevention studies

Intervention coding	Intervention name	Intervention duration + frequency	Intervention description	Study author (year)	Study Design	Population
**Resilience training**	Trauma prevention training program	90-min sessions over 5 days	Program was designed to strengthen resilience during stressful encounters and teach methods of coping after exposure, thereby preventing the emergence of maladaptive symptoms and behaviors with adverse effects on professionalism.	Arble, E (2017) [31]	Quasi-experimental	Police
	Police trauma resilience training	2-h training once/week for 10 weeks	An initial psycho-educational session, followed by ten small group sessions consisting of relaxation and imagery training with mental skill rehearsal. Twelve months later, psychophysiological stress and police work performance were assessed during a live critical incident simulation.	Arnetz, BB (2009) [32]	RCT	Police
	Mindfulness-based resilience training	2-h class once/week for 8 weeks	A curriculum designed to train participants in a number of experiential exercises evoking qualities of mindfulness: mental focus, sustained attention, and a broad sense of personal and situational awareness. These exercises include versions of the body scan (body awareness exercise), sitting meditation, mindful movement, walking meditation, eating meditation, mindful martial arts exercises, and other elements of mindfulness-based stress reduction (MBSR). During class, there are periods of discussion exploring the experience of these exercises, as well as daily homework practice of the experiential mindfulness exercises, supplemented with several readings and journaling.	Christopher, MS (2016) [33]	Longitudinal cohort study	Police
	Visuo-motor behavior rehearsal	10 and 20 min consecutively	Progressive relaxation portion and an imagery/mental rehearsal portion prior to undergoing a highly stressful, critical event training scenario involving “live fire.”	Shipley, P (2002) [34]	RCT	Police
**Stress management**	Stress management program	4 h/week for 6 weeks	The program included physiological and psychological interventions, such as relaxation training, physical exercise, dietary advice, rational emotive modeling, assertiveness/communication training.	Ackerley, DG (1986) [35]	RCT	Police
	Stress reduction program	2.5 h × 4 sessions	The program utilized a cognitive-behavioral approach to training to teach stress awareness and stress control, including an assessment of current factors present in the policeman’s life which are stressful to him, a presentation of general information commonly used in stress management, and didactic interchange with class participants utilizing specific stressors listed by those participants.	Coulson, JE (1987) [36]	Quasi-experimental	Police
	HeartMath® stress and emotional self-management training	4–6 h × 3 sessions over 1 month	Program provided practical techniques designed to reduce stress in the moment, improve physiological and emotional balance, increase mental clarity, and enhance performance and quality of life.	McCraty, R (1999) [37]	RCT	Police
	Stress management training	8-h session	Stress awareness training and stress management training.	Oliver, WM (2009) [38]	Observational	Police
	Stress management training	2-h sessions × 6	Stress awareness training and stress management training, with key components including self-monitoring in stressful situations, muscle relaxation, and development of adaptive self-statements.	Sarason, IG (1979) [39]	RCT	Police
	Stress inoculation training	2-h sessions × 5 over 7 weeks	Three major components which generally followed the phases of (1) conceptualization, (2) skills acquisition and rehearsal, and (3) application and follow-through.	Digliani, JA (1994) [40]	RCT	Police
	Cognitive-behavioral stress management	4 weekly sessions over 1 month	Key components include stress and trauma education, relaxation techniques, problem-solving, and communication skills. In the therapeutic part of the program, CBT techniques were used, while in the educational part of the program, stress and trauma-related issues were the focus.	Sijaric-Voloder, S (2008) [41]	RCT	Police
**Suicide prevention**	Suicide prevention program	6 months of presentations and online course	The program included (1) suicide awareness training, (2) suicide prevention training, and (3) education training for crisis management.	Finney, EJ (2015) [42]	Quasi-experimental	Firefighters
	Badge of life psychological survival for police officers program/emotional self-care training program	NR	Suicide prevention program including awareness training, further awareness training materials available online to facilitate face-to-face delivery where preferred, annualized mental health “check-ups” with mental health professionals, and peer support programming.	Levenson, RL Jr (2010) [43]*	Descriptive	Police
	Together for life program	NR	The program involved training for all officers, supervisors, and union representatives as well as establishing a volunteer helpline and a publicity campaign.	Mishara, BL (2012) [44]	Quasi-experimental	Police
	Suicide prevention program	NR	This program included awareness training, gatekeeper training, a 24-h crisis telephone hotline, life skills and stress management workshops, a crisis intervention team, suicide postvention services, and changes to media reporting guidelines following the suicide of an officer.	Welch, J (1998) [45]	Quasi-experimental	Police
**Other health promotions**	Power to change performance program	5 modules over 2 days (positive emotion techniques); daily physiological readings	The program focused on stress and health risk reduction, including a positive-emotion refocusing technique with physiological feedback training (heart rhythms).	McCraty, R (2003) [46]	RCT	Correctional
	Health assessment and promotion program	5–15 min each	Health assessment and motivational interviewing intervention, plus self-help materials for alcohol, smoking, and stress, an advice booklet, and audio cassette.	Richmond, RL (1999) [47]	RCT	Police
	Safety & Health Improvement: Enhancing Law Enforcement Departments (SHIELD) wellness intervention	30-min sessions once/week for 12 weeks	Team-based intervention that fosters social support and accountability; each member of the team would discuss weekly goals and there was a scripted set of questions to answer out loud regarding successful strategies identified by subjects to reach the weekly goal.	Kuehl, KS (2016) [48]	RCT	Police
	Health promotion through fitness training	30–40 min/session, 3×/week for 10 weeks	Aerobic (i.e., running) and anaerobic (i.e., weight training) structured group sessions. Intervention groups were encouraged to do unsupervised sessions at home when they missed sessions.	Norris, R (1990) [49]	NRCT	Police
	Circuit weight training program	20 min/session, 3×/week for 16 weeks,	Circuit weight training, exercises, and sets of reps. Proper technique and individualized training guide provided.	Norvell, N (1993) [50]	RCT	Police
	Education and aerobic conditioning	90-min sessions over 8 weeks (education); 3× 45-min sessions/week for 8 weeks (aerobic conditioning)	Exercise/fitness education and aerobic conditioning sessions.	Short, MA (1984) [51]	RCT	Police
	Yoga program	75-min classes 6×/week for 4 non-continuous weeks	Classes involved Kripalu yoga (focusing on mindfulness, deep relaxation, yoga postures, meditation, and breathing).	Jeter, PE (2013) [52]	Quasi-experimental	Police
	Educational sessions with sleep screening	30-min (mandatory educational sessions)	The program included (1) mandatory educational sessions for sleep, (2) voluntary sleep disorders screening, and (3) sleep disorders diagnosis and treatment for those who screened at risk for a sleep disorder.	Sullivan, JP (2017) [53]	RCT	Firefighters
	Sleep hygiene training	NR	Workshop on self-management techniques for controlling insomnia, with the intention of heightening subjects’ awareness and increasing their knowledge of sleep hygiene.	Holbrook, MI (1994) [54]	Quasi-experimental	Police

*CBT* cognitive-behavioral therapy, *NR* not reported, *RCT* randomized controlled trial

*Levenson et al. (2010) focused on describing the badge of life training program and did not measure any outcomes

**Table 4:** Interventions for rehabilitation studies

Intervention coding	Intervention name	Intervention duration/frequency	Intervention description	Study author (year)	Study design	Population
Drug therapy	Carbemazepine and sodium valproate	500 mg 2× daily (sodium valproate)	The patient first received carbemazepine added to the other medications, then withdrew due to side effects. The patient was re-admitted to the hospital and sodium valproate was commenced. After the symptoms improved, the patient was discharged and followed up as an outpatient.	Ford, N (1996) [55]	Case report	Police
	Prazosin	1 mg once/day for 1 week, then increase by 1 mg every 3-4 days thereafter, to up to 6 mg after 4 weeks	Prazosin administered to the patient with increasing dosages.	Coupland, NJ (2009) [56]	Case report	Firefighters
Psychotherapy	Brief eclectic psychotherapy (BEP)	60-min sessions once/week for 16 weeks	Combines cognitive-behavioral and psychodynamic approaches (including 5 essential elements: psycho-education, imaginary guidance, writing assignments and mementos, domain of meaning or integration, and a farewell ritual) over sessions of individual psychotherapy.	Gersons, BPR (2000) [57]	RCT	Police
	Brief eclectic psychotherapy (BEP)	16 weekly sessions	Combines cognitive-behavioral and psychodynamic approaches (including 5 essential elements: psycho-education, imaginary guidance, writing assignments and mementos, domain of meaning or integration, and a farewell ritual) over sessions of individual psychotherapy.	Plat, MCJ (2013) [58]	Observational	Police
	Brief psychological intervention	1.5-h session	A group intervention was given within 24 h after a critical incident. Briefly, participants described what they had done, expressed what they felt at the time of the incident, and talked about any symptoms. The therapist explained typical reactions and the normality of feeling anger, guilt, and having nightmares. Participants related past experience to the current incident. The therapist then summed up what the participants had expressed.	Bohl, N (1991) [59]	NRCT	Police
	Brief psychological intervention	1.5-h session	A group intervention was given within 24 h after a critical incident. Briefly, participants described what they had done, expressed what they felt at the time of the incident, and talked about any symptoms. The therapist explained typical reactions and the normality of feeling anger, guilt, and having nightmares. Participants related past experience to the current incident. The therapist then summed up what the participants had expressed.	Bohl, N (1995) [60]	NRCT	Firefighters
	Cognitive-behavioral treatment	60-min sessions × 15 over 7 months	The treatment included building rapport, development of alternate and adaptive mechanisms for coping, progressive muscle relaxation, introducing of assimilation and rational thinking as coping mechanisms, gradual exposure to the traumatic events with discussions of trauma, as well as relapse prevention and review of progress.	Cornelius, TL (2007) [61]	Case report	Police
	Individual wellness counseling sessions	60 min every other week × 5 (cognitive-behavioral personalized wellness counseling) 5 sessions every other week over 10 weeks (individual counseling)	Cognitive-behavioral counseling personalized wellness sessions every other week. Referral to mental health services as required.	Tanigoshi, H (2008) [62]	RCT	Police
	Critical incident stress debriefing	NR	A peer counseling group procedure with psychoeducational components that provide information on various stress reactions following exposure to a critical incident. The strategy in this group meeting is to begin with materials that the participants are comfortable in discussing, leading to more emotionally intense exchanges, and concluding with a psychoeducational component intended to bring closure to the group. Strategy uses 7 stages: (a) introduction, (b) fact, (c) thought, (d) reaction, (e) symptoms, (f) teaching, and (g) reentry.	Harris, MB (2002) [63]	Observational/cohort	Firefighters
	Critical incident stress debriefing (CISD)	Within 72-hr after a critical incident	A peer counseling group procedure with psychoeducational components that provide information on various stress reactions following exposure to a critical incident. The strategy in this group meeting is to begin with materials that the participants are comfortable in discussing, leading to more emotionally intense exchanges, and concluding with a psychoeducational component intended to bring closure to the group. Strategy uses 7 stages: (a) introduction, (b) fact, (c) thought, (d) reaction, (e) symptoms, (f) teaching, and (g) reentry.	Leonard, R (1999) [64]	Observational	Police
	Critical incident debriefs	NR	Group-based debriefing sessions.	Ruck, S (2013) [65]	NRCT	Correctional
	Critical incident stress debriefing (CISD)	~ 90 min, within 72-h after a critical incident	A peer counseling group procedure with psychoeducational components that provide information on various stress reactions following exposure to a critical incident. The strategy in this group meeting is to begin with materials that the participants are comfortable in discussing, leading to more emotionally intense exchanges, and concluding with a psychoeducational component intended to bring closure to the group. Strategy uses 7 stages: (a) introduction, (b) fact, (c) thought, (d) reaction, (e) symptoms, (f) teaching, and (g) reentry.	Tuckey, MR (2014) [66]	RCT	Firefighters
	Individual debriefing	3 sessions in total at 24-h, 1 month, 3 months	The debriefer applies a seven-stage, semi-structured procedure, comprising of: an introduction, facts, thoughts and impressions, emotional reactions, normalization and traumatic stress education, planning for the future, and disengagement.	Carlier, IVE (2000) [67]	NRCT	Police
	Eclectic group counseling	1.5–2-h sessions once/week for 3 months	Counseling included the interactive model of cognitive-behavioral therapy, religious interventions, mandala drawing, and reality therapy.	Chongruksa, D (2012) [68]	RCT	Police
	Crisis debriefing groups	1 single session	A single session for relieving the distress of emergency service workers encountering traumatic events in the line of duty.	Regehr, C (2001) [69]	Observational	Firefighters
	Exposure-based therapy and cognitive restructuring	NR	Psychotherapy (i.e., exposure-based therapy and cognitive restructuring, or ETCR) for police officers with partial post-traumatic stress disorder (pPTSD).	Peres, JFP (2011) [70]	NRCT	Police
	Trauma risk management (TRiM)	NR	Peer support intervention using trained, non-medical personnel to conduct a psychological risk assessment for those exposed to potentially traumatic events. TRiM interviews can be delivered to individuals (a 1:1 intervention) or within small groups; the police service currently uses mostly 1:1 interventions.	Hunt, E (2013) [71]	Cohort study	Police
	Trauma risk management (TRiM)	NR	NR	Watson (2014) [72]*	NR	NR
Other therapies	Exposure therapy	90-min weekly sessions × 5	Therapy sessions involving imaginal exposure (e.g., deliberately recounting the trauma) and in vivo exposure (e.g., exposure to stimuli that remind the patient of past trauma).	Tolin, DF (1999) [73]	Case report	Police
	Eye movement desensitization and reprocessing (EMDR)	5–6 sessions	Psychological treatment for post-traumatic stress disorder (PTSD).	Kitchiner, NJ (2004) [74]	Case report	Firefighters
	Eye movement desensitization and reprocessing (EMDR)	2–3-h sessions conducted 3–4 weeks apart	Psychological treatment for PTSD. Subjects were taught coping and “containment” techniques, how to identify and develop support networks, and how to log their trauma-related memories—a necessary precondition for eye movement desensitization and reprocessing (EMDR). The first (pre-EMDR) brain SPECT scans were collected before EMDR took place. This procedure gave bilateral stimulation in the subjects’ palms and fingers, thus allowing them to re-experience traumatic scenes. This was followed by a “reconciliation phase” of treatment, focusing on the re-scripting of relational patterns that might not have been corrected once subjects became detraumatized.	Lansing, K (2005) [75]	Observational	Police
	Eye movement desensitization and reprocessing (EMDR)	2-h EMDR sessions × 3	Psychological treatment for PTSD. The EMDR sessions took place off-site at the office of the therapist assigned to the officer. The stressors identified in the clinical interview served as the focus of the EMDR sessions.	Wilson, SA (2001) [76]	RCT	Police
	Writing intervention	15-min writing once/day for 4 consecutive shifts	Emotional disclosure in writing as a coping method for police officers; they received instructions to write about their strong emotions, positive or negative, related to work or not, and about what they plan to do, if anything, as a result of the emotions.	Ireland, M (2007) [77]	RCT	Police

*BEP* brief eclectic psychotherapy, *CISD* critical incident stress debriefing, *EMDR* eye movement desensitization and reprocessing, *PTSD* post-traumatic stress disorder, *NR* not reported, *NRCT* non-randomized controlled trial, *RCT* randomized controlled trial, *TRiM* trauma risk management

*Watson (2014) was an unpublished thesis; information was extracted from the review only

#### Prevention interventions

Prevention strategies were explored in 24 primary studies. Twenty-one of these studies included police (officers, recruits, veterans, management and support staff), 2 focused on firefighters, and 1 study was conducted with correctional staff.

##### Suicide prevention (*n* = 4 studies)

Four studies examining suicide prevention programs involving various interventions were included. The Together for Life program (Mishara et al., 2012) [44] was a comprehensive suicide prevention strategy targeting the Montreal Police Force. The intervention involved training for all officers, supervisors, and union representatives, as well as establishing a volunteer helpline and a publicity campaign. Twelve years after implementing the program, the study reported a statistically significant decrease in suicide rates in Montreal police (− 78.9%; *p* = 0.008) and a statistically significant reduction in suicide rates post-program compared to police in other provinces (*p* = 0.007). Another suicide prevention program for police officers described by Welch and colleagues [45] in South Africa also found reduced depression, PTSD, and suicide numbers post-intervention; however, statistical significance was not tested. Similarly, a report was published describing the Badge of Life Psychological Survival for Police Officers Program (BOL); however, the effectiveness of this program on suicide rates was not evaluated [43]. Finally, Finney et al.’s [42] quasi-experimental study found no statistically significant differences in suicide rates after the implementation of a suicide prevention program to educate firefighters about suicide.

##### Resilience training (*n* = 4 studies)

Two studies explored effective resilience programs in junior officers. Arble et al. [31] examined a program designed to increase resilience and build coping mechanisms while reducing trauma. Each session in the quasi-experimental study used audio recordings and scripts to take new police officers through a sequence of imagined scenarios beginning with a relaxation scenario and building up to an enhanced trauma scenario. After the program, a statistically significant increase in the use of positive coping strategies like humor (mean change = 0.78; *p* = 0.02) and positive reframing (0.61; *p* = 0.02), as well as statistically significant decreases in anxiety (− 0.42; *p* = 0.02) and substance abuse (− 0.39; *p* = 0.04) were reported in the new officers. Similarly, in a randomized trial [32], a group of rookie police officers (i.e., 1 year of experience on the Swedish police force) with trauma resilience training was compared to a group that received regular training. The resilience training involved education sessions, imagery training, and mental skills rehearsal and resulted in statistically significantly less negative mood (mean change = − 1.11; *p* = 0.03), but no change in positive mood when compared to the control officers.

Mindfulness-based resilience training (MBRT) was assessed in a longitudinal cohort study by Christopher et al. [33]. The 8-week training program used experiential exercises like body awareness scans, meditation, martial arts, and mindful movement to introduce police officers to the practice of mindfulness. Group classes were supplemented with reading material and take-home practice exercises. In this study, improvement was seen during the program in many outcomes including a statistically significant increase in resilience (mean change = 0.7, *p* = 0.001), mindfulness (1.19; *p* < 0.001), mental health (0.78; *p* < 0.001), emotional intelligence (0.74; *p* = 0.01), and physical health (0.48; *p* = 0.04), and statistically significant decreases were noted in fatigue (0.59; *p* < 0.001), anger (0.63; *p* < 0.001), burnout (0.74; *p* = 0.001), difficulties with emotional regulation (0.83; *p* = 0.01), perceived general stress (0.75; *p* < 0.001), organizational stress (0.72; *p* = 0.002), and operational stress (0.56; *p* = 0.007). Visuo-motor behavior rehearsal (VMBR) is a sequenced simulation intervention involving a relaxation period, followed by an imagery rehearsal opportunity, and ending with a highly stressful, critical event-training scenario. Shipley et al. [34] found that participants in the VMBR group experienced statistically significantly lower anxiety (mean difference = 1.72; *p* < 0.05) when compared to the control group as measured by the cognitive state anxiety component of the Competitive State Anxiety Inventory-2 (CSAI-2) scale, but no significant differences were reported in the other two components of this scale.

##### Stress management (*n* = 7 studies)

Four randomized trials were conducted that evaluated stress management programs for police officers. HeartMath was a program involving 3 training sessions aimed at reducing stress, improving physiological and emotional balance, increasing mental clarity, and enhancing performance and quality of life. McCraty et al. [37] reported improvements in some of these areas including considerable decreases in fatigue and the overall global negative emotion subscale; the latter subscale is the average of the individual scores for the anger, distress, depression, and sadness constructs based on the Personal and Organizational Quality Assessment survey [37]. Sijaric-Voloder et al. [41] also reported on the development and evaluation of a stress management program for police officers involving 4 sessions a week over 4 weeks covering stress and trauma awareness, relaxation training, problem-solving skills, and communication techniques. The program resulted in a statistically significant reduction on the Beck Anxiety Inventory (*p* value not reported) and the Anxiety Sensitivity Index (*p* value not reported) scales, as well as a non-significant improvement in coping strategies, somatic reactions, and job performance when compared to the control. Six 2-h sessions involving self-monitoring training, relaxation techniques, and building coping responses comprised a stress management program by Sarason et al. [39]. The authors found statistically significant improvements in performance for the group receiving training compared to the control across a range of simulated situations; however, no differences in stress outcomes were found. A fourth stress management program (4 h/week for 6 weeks) involving relaxation techniques, exercise, and diet advice, as well as rational emotive modeling, and assertiveness/communication strategies resulted in no statistically significant differences in sick leave, burnout, stress, or coping outcomes compared to the control group [35].

An in-service stress management training session was described in an observational study by Oliver et al. [38] involving 4 h of stress awareness and recognition training, followed by 4 h of management training. Self-reported job stress levels were statistically significantly reduced at 18 months (*t* = 15.272; *p* < 0.0041) and although there was a statistically significant decrease in anxiety over the first 12 months (2.215; *p* < 0.0041), eventually, anxiety and behavioral scales scores increased over time. Two additional studies described a stress reduction program, one using a cognitive-behavioral approach to training [36] and another using stress inoculation training comprised of conceptualization, skills acquisition and rehearsal, application, and follow-through phases [40], with no statistically significant differences in mental health outcomes found in either.

##### Other health promotion (*n* = 9 studies)—physical, mental, and emotional health/education

One study examined the SHIELD (Safety & Health Improvement: Enhancing Law Enforcement Departments) program, a wellness, team-based intervention with the aim of reducing occupational risks and unhealthy behaviors in police officers [48]. The intervention involved weekly sessions on healthy eating, exercise, weight, stress, and sleep, with an emphasis on team social support. The study reported statistically significant favorable changes with respect to sleep (0.2; *p* < 0.05), stress (0.13; *p* < 0.05), systolic blood pressure (0.13; *p* < 0.10), tobacco use (0.09; *p* < 0.05), and alcohol consumption (0.12; *p* < 0.10) in officers enrolled in the program; however, of these, only alcohol/tobacco reductions were sustained overtime.

The impact of physical health promotion in police officers was examined in 4 studies. In the Short et al. [51] study, obese police officers were randomized to an instruction group that involved 8 weekly 90-min sessions covering exercise and nutrition or to a conditioning group that received the above instruction as well as a program of walk-jog activities for up to 45-min, 3 times a week. Both groups had increases in positive psychological symptoms reported using the Tennessee Self-concept Scale (TSCS), including physical self (conditioning mean change = 6.27; *p* < 0.01, instruction mean change = 2.84; *p* < 0.05) and self-satisfaction (conditioning = 6.46; *p* < 0.01, instruction = 2.39; *p* < 0.05) with the conditioning group increasing significantly more over time. Norris et al. [49] conducted a 3-arm study comparing structured aerobic group exercise sessions, structured anaerobic group sessions, and no exercise sessions. The aerobic group showed a statistically significant decrease in psychological symptoms of ill health (*F* = 8.69; *p* < 0.001), heart rate (37.25; *p* < 0.001), timed run (52.98; *p* < 0.001), blood pressure (systolic 8.47, *p* < 0.001; diastolic 22.69, *p* < 0.001), and job stress (5.08; *p* < 0.01), as well as a statistically significant increase in quality of life (24.38; *p* < 0.001) when compared to control. Another physical fitness intervention including circuit weight training was studied by Norvell et al. [50]. Four months of circuit weight training in the intervention group resulted in statistically significant positive effects on the Perceived Stress Scale (*F* = 7.39; *p* < 0.01), the Cohen-Hoberman Inventory of Physical Symptoms (25.77; *p* < 0.001), and the Hopkins Symptom Checklist-90 Global Severity Index including subscales for anxiety, depression, and hostility in comparison to the control group (32.54; *p* < 0.001). In a fourth study [52], yoga was incorporated in a police academy training program to assess the impact on stress, mood, and mindfulness on police recruits. After 6 yoga classes, the study found a statistically significant reduction in perceived stress (mean difference = − 1.44 (SD 3.89); *p* < 0.05) and increase in mood (8.85 (SD 14.63); *p* < 0.05). When surveyed, some trainees found the program to be beneficial and relaxing, while others were resistant to the idea of yoga as a part of police training.

A sleep health education program for firefighters was described by Sullivan et al. [53]. The treatment group had statistically significantly fewer disability days and was less likely to file an injury report during the study than the control group (*F* = 8.79; *p* = 0.003).

One study [46] assessed the use of a prevention program in correctional officers. The Power to Change Performance program involved 5 training modules over 2 days focusing on positive emotion re-focusing techniques. After the program, the group receiving the intervention reported a statistically significant reduction in fatigue (mean change = − 0.37(1.16); *t* = 2.03; *p* < 0.05), hostility (− 0.15(0.43); *t* = 2.13; *p* < 0.05), feelings of inadequacy and self-doubt (− 0.19(0.55); *t* = 2.16; *p* < 0.05), paranoid ideation (− 0.17(0.50); *t* = 2.19; *p* < 0.05), DHEA (dehydroepiandrosterone) levels (− 2.45(1.82); *t* = 6.33; *p* < 0.001), and overall psychological distress (− 4.53(11.11); *t* = 2.57; *p* < 0.05). The control group also had a statistically significant increase in depression (mean change = 0.24(0.49); *t* = − 2.47; *p* < 0.05).

Holbrook et al. [54] assessed a 1-h education workshop on self-management to improve sleep hygiene. Despite statistically significant increases in awareness of sleep hygiene (*t* = 9.23; *p* < 0.001) and knowledge about nicotine (4.24; *p* < 0.001), hypnotics (4.64; *p* < 0.001), and caffeine (7.53; *p* < 0.001) immediately post intervention, these differences were not significant one month after the workshop. Finally, Richmond et al. [47] studied a brief health assessment and motivational interviewing intervention aiming to reduce excessive drinking, smoking, and stress among police. The study reported some positive trends in both groups overtime; however, no statistically significant differences were found between the intervention and control groups.

#### Rehabilitation interventions

A total of 23 studies reported rehabilitation strategies and programs, including 16 targeting police officers, 6 targeting firefighters, and 1 focusing on correctional officers.

##### Psychotherapy (*n* = 16 studies)

Sixteen studies described the use of psychotherapy in the rehabilitation of police officers (11), firefighters (4), and correctional staff (1). Debriefing sessions, including critical incident stress debriefing (CISD), a 7-phase counseling intervention employed after a crisis or critical event, was examined in 8 studies. In police officers, Bohl [59] reported statistically significantly lower negative psychological health outcomes, such as depression (*t* = 4.18; *p* < 0.01), anger (2.42; *p* < 0.02), and long-term stress (6.77; *p* < 0.01) in the CISD group in comparison to the control group receiving no intervention, but no difference in anxiety was found between the groups. Leonard et al. [64] found that CISD resulted in statistically significantly lower levels of anger (i.e., state anger *t* = 3.35, *p* < 0.001; trait anger 2.27, *p* < 0.05; angry temperament 3.04, *p* < 0.01) and increased the use of some adaptive coping techniques (i.e., active coping *F* = 4.50, *p* < 0.05; positive reinterpretation and growth 7.26, *p* < 0.01); however, there was no significant overall difference in coping between the CISD and control groups. Finally, in another study [67], 3 successive individual CISD sessions conducted after experiencing a critical event resulted in statistically significantly more re-experiencing of PTSD symptoms in the debriefed group compared to the control group 1 week post-trauma (post treatment = 38; post control = 21; *p* < 0.01), and no statistically significant difference in psychological morbidity between groups at 6 months.

In firefighters, CISD compared to control resulted in statistically significant positive psychological health outcomes (anxiety *t* = 9.81, *p* < 0.001; depression *t* = 7.74, *p* < 0.001; and long-term stress *t* = 5.5, *p* < 0.001) in one study [60]; however, another found no statistically significant relationship between CISD and PTSD symptoms [63]. Firefighters in a third study receiving CISD [66] reported statistically significant lowered alcohol consumption (*F* = 4.78; *p* < 0.05) and a trend towards increased quality of life, but no effects on psychological distress or post-traumatic stress were found. Regehr et al. [69] studied the use of a modified crisis debriefing intervention in Australian firefighters, and although the majority perceived the debriefings to be helpful, the study did not report statistically significant changes in psychological outcomes. Prison guards, in a study conducted by Ruck et al. [65], were offered an adapted CISD program after experiencing traumatic workplace events. Those who accepted debriefing experienced a statistically significant reduction in anxiety (*F* = 0.01, effect size = 0.06; no *p* value given), depression (*F* = 0.01, effect size = 0.02; no *p* value given), and PTSD (*F* = 36.8, effect size = 0.62; *p* < 0.01).

Three studies reported on the use of cognitive-behavioral therapy (CBT). A case study of a retired police officer [61] found a CBT exposure-based approach resulted in overall improvement in psychological symptoms, but no test of significance was conducted. A study assessing a wellness counseling program with elements of CBT reported higher wellness scores (Wilks’s lambda = 0.81, *F* = 11.76; *p* = 0.001) in the counseling groups between pre- and post-treatment [62]. A study described a group counseling intervention that combined CBT with religious interventions, mandala drawing, and reality therapy [68]. In this study, the group counseling intervention was targeted to police officers in terrorist situations and found statistically significantly favorable effects in general health (*F* = 15.27, *η* = 0.276, *p* = 0.000), depression (*F* = 5.33, *η* = 0.118; *p* = 0.026), and overall psychological symptoms (*F* = 5.83, *η* = 0.127; *p* = 0.020) including anxiety, hostility, and paranoia at 1 month post-intervention.

Brief eclectic psychotherapy (BEP), a combination of CBT and other psychotherapeutic elements, was used to treat PTSD in police officers in two studies. In the Gersons et al. [57] study, BEP showed a statistically significant improvement in all PTSD-related outcomes, psychological symptoms, and return to work measures when compared to the control group, but no difference in symptoms listed on the Anxiety Disorders Interview schedule. A retrospective analysis of a BEP protocol [58] found that of 59 officers on sick leave due to PTSD, 48 returned to work post-intervention. A third study investigated exposure-based therapy and cognitive restructuring (ETCR) in a group of police officers who experienced a gunfire attack [70], where the therapy group experienced significantly decreased PTSD symptoms after ETCR (pre mean 48 (3.62), post mean 19 (5.03); *p* = 0.03).

Trauma risk management (TRiM) is a post-trauma psychological risk assessment to identify those at high risk of developing negative psychological symptoms after a trauma. Two studies [71, 72] examined the use of this program in a police force. Both found that TRiM may play a role in identifying and providing early intervention to those experiencing trauma and lowering psychological distress.

##### Drug therapy (*n* = 2 studies)

Two case reports examined the impact of drug therapy. Coupland [56] found that 1 mg of prazosin helped reduce insomnia and nightmares in a firefighter with PTSD, and carbamazepine and sodium valproate improved PTSD symptoms, including sleep and depression in a police officer [55]. Neither report tested for significance.

##### Other therapies (*n* = 5 studies)

Three studies reported on the effects of eye movement desensitization and reprocessing (EMDR) on firefighters and police officers who had PTSD. Six police officers undergoing EMDR therapy had statistically significant reductions in mean PTSD symptoms (pre mean 43.2, post mean 5.2; *p* = 0.003) in comparison to the control group [75]. Wilson et al. [76] conducted an RCT of 62 police officers comparing the effects of EMDR with a stress management program on PTSD and stress symptoms. This study found that EMDR was statistically significantly more effective in lowering PTSD symptoms (*F* = 4.45; *p* < 0.05) and stress (7.47; *p* < 0.05) in comparison to the stress management program. Finally, Kitchiner [74] presented individual case reports where EMDR therapy was generally helpful in reducing PTSD symptoms in firefighters; however, no test of significance was conducted.

One case study examined the effects of exposure therapy, a type of therapy in which the subject is made to face stimuli or memory of a trauma, on a police officer with PTSD [73]. This therapy was administered over 5 weekly sessions lasting 90 min and the participant reported long-term relief of PTSD symptoms, even 6 months after the intervention was complete. Finally, an RCT conducted by Ireland et al. [77] examined the effect of writing about personal emotions on the distress level of police officers. Police officers were required to write for 15 min, 4 days a week. The writing therapy group had statistically significantly lower levels of anxiety (*p* = 0.001), depression (*p* = 0.047), and stress (*p* = 0.002) in comparison to police officers who did not participate in writing therapy.

## Discussion

### Summary of evidence

We conducted a rapid overview to identify interventions targeting the prevention and management of OSI among frontline community safety personnel for the Ministry of Community Safety and Correctional Services of Ontario. In our initial scoping of the literature, we found no previous overviews conducted on this important topic, but several reviews existed. This overview included 14 relevant reviews, which contained 47 unique primary studies. While the reviews contained 7 to 43 studies each, very few of these studies evaluated interventions, and those that did varied significantly in study design and outcomes reported. These differences meant we were unable to combine the study results in a meaningful way using meta-analyses; therefore, results were summarized descriptively. The majority of the primary intervention studies were conducted in police populations, followed by firefighters. Only two intervention studies were targeted towards correctional officers and no intervention studies were found for coroners and/or forensic pathologists, highlighting important gaps in the literature for these populations.

Results of the primary studies show some promising prevention strategies in police officers, specifically, resilience training programs and other health promotion strategies, including a combination of physical, mental, and emotional education. Suicide prevention programs as well as stress management programs showed mixed results in reducing suicide rates and other psychological outcomes, with the majority of studies failing to report statistical significance.

Psychotherapy was the largest group of rehabilitation strategies included in this overview with varied results in police, firefighters, and correctional officers. Debriefing and CISD significantly reduced depression, anger, stress, and alcohol consumption in some studies but no significant differences were reported in others, and the one study assessing the effect of CISD in prison guards found improvements in depression, anxiety, and PTSD outcomes. Other psychotherapy interventions including BEP, ETCR, CBT, and TRiM, as well as drug therapy interventions, EMDR, and writing therapy, may also be effective in treating symptoms of OSI.

The mixed results from these primary studies are in line with the results of the reviews included in our rapid overview, as well as reviews conducted in other populations experiencing OSI. Two reviews examining mind and body therapy in military veterans showed improvement in PTSD symptoms and overall health of the participants [78, 79], while a recent review of suicide prevention interventions in veterans found inconclusive results leading the study authors to recommend additional exploration using refined methods [80]. The similarity across populations further highlights the need for robust studies to better serve those impacted by OSI.

### Strengths and limitations

There were several notable strengths of this overview, including an a priori design by means of a protocol registered on PROSPERO (CRD42019125945). The guidelines set forth by the Cochrane Handbook [10, 11] were used to conduct this overview, in addition to the AMSTAR 2 tool [16] for assessing the quality of included reviews. Finally, all screening and data abstraction of reviews was done in duplicate with a calibration exercise completed prior to every step, to ensure reviewer consistency.

Although efforts were made to conduct a methodologically rigorous overview, there were some unavoidable limitations. The major limitation was the time constraint to meet the needs of the knowledge user, allowing only for an overview of reviews as opposed to a systematic review and meta-analysis. Thus, only primary studies found in published reviews were included and potentially relevant primary studies not contained in systematic reviews would not have been captured in our synthesis. In addition, to ensure a “rapid” overview process, limits were placed on our search strategy (i.e., English-language published in the past 10 years), quality appraisal of the included reviews and abstraction of study-level data was completed by one person and verified by another, and the quality of the individual primary studies summarized in our overview was not appraised. As a result, although this overview was the first to identify a broad range of interventions in the literature for frontline community safety personnel, our results should be interpreted with caution.

### Conclusion

The results from this overview suggest that potentially effective prevention and rehabilitation strategies exist targeting first responders at high-risk of developing OSI. However, further investigation is needed before the interventions can be implemented within specific first responder populations, especially correctional service workers and coroners. Our findings will serve as a basis for the MCSCS to develop an evidence-based strategy to tackle OSI in frontline community safety personnel and first responders. The suggested next step would be to conduct a systematic review of primary studies to help inform the development and examination of interventions targeted to this population.

## Supplementary information

**Additional file 1.** The additional file includes the completed PRISMA PRIO-harms checklist, MEDLINE search strategy, matrix of study overlap, AMSTAR 2 appraisal results of included reviews, summaries of police, firefighter and correctional officer primary studies, and a list of excluded primary studies.

## Data Availability

All datasets used and/or analyzed during this study are included in this published article.

## Abbreviations

AMSTAR 2: A Measurement Tool to Assess Systematic Reviews (version) 2
BEP: Brief eclectic psychotherapy
CBT: Cognitive-behavioral therapy
CI: Confidence interval
CISD: Critical incident stress debriefing
EMDR: Eye movement desensitization and reprocessing
EMS: Emergency medical services
ETCR: Exposure-based therapy and cognitive restructuring
MBRT: Mindfulness-based resilience training
MCSCS: Ministry of Community Safety and Correctional Services, Ontario, Canada
NRCT: Non-randomized controlled trial
OSI: Occupational stress injury or illness
PRISMA: Preferred Reporting Items for Systematic Reviews and Meta-Analyses
PTSD: Post-traumatic stress disorder
RCT: Randomized controlled trial
SD: Standard deviation
TRiM: Trauma risk management
UK: United Kingdom
USA: United States
VMBR: Visuo-motor behavior rehearsal
