# Supplementary material for: Interventions for the prevention and management of occupational stress injury in first responders: a rapid overview of reviews
Source: Syst Rev. 2020 May 31;9:121. doi: 10.1186/s13643-020-01367-w (PMC7262749; doi:10.1186/s13643-020-01367-w)
Supplement: Supplementary file 1 — Additional file 1. The additional file includes the completed PRISMA PRIO-harms checklist, MEDLINE search strategy, matrix of study overlap, AMSTAR 2 appraisal results of included reviews, summaries of police, firefighter and correctional officer primary studies, and a list of excluded primary studies. [file 13643_2020_1367_MOESM1_ESM.docx]

**Additional File 1**

[Appendix A: PRIO-harms Checklist 2](#_Toc37274445)

[Appendix B: Database Search Strategy 6](#_Toc37274446)

[Appendix C: Overlap table 9](#_Toc37274447)

[Appendix D: Quality appraisal of included reviews (AMSTAR-2 Results) 12](#_Toc37274448)

[Appendix E: Details of included primary studies 14](#_Toc37274449)

[Table E1. Police studies 14](#_Toc37274450)

[Table E2. Firefighter studies 26](#_Toc37274451)

[Table E3. Correctional officer studies 30](#_Toc37274452)

[Appendix F: List of excluded primary studies 31](#_Toc37274453)

# Appendix A: PRIO-harms Checklist

| **Section/Topic** | **(Sub-) item #** | **Checklist item** | | | | | **Reported**  **on page #** |
| --- | --- | --- | --- | --- | --- | --- | --- |
| **TITLE** | | | | | | |  |
| 1. Title | 1a | Specify the study design with terms such as “overview of (systematic) reviews,” “umbrella review,” “(systematic) review of systematic reviews,” or “(systematic) meta-review” in the title of the OoSRs. | | | | | 1 |
|  | 1b | Mention “safety” or harms related terms, or the adverse event(s) of interest in the title of the OoSRs. | | | | | NA |
| **ABSTRACT** | | | | | | |  |
| 2. Structured-like summary | 2a | Provide a structured-like abstract, as applicable: background, objective, data sources, selection criteria, data extraction, review appraisal, data synthesis methods, results, limitations, conclusions. | | | | | 3 |
|  | 2b | Report the main findings of analysis of harms undertaken in the OoSRs or/and in the included SRs. | | | | | NA |
| **INTRODUCTION** | | | | | | |  |
| 3. Rationale | 3a | Specify the rationale and the scope (wide or narrow agendas) for the overview in the context of an existing body of knowledge on the topic. | | | | | 5-6 |
|  | 3b | Provide a balanced presentation of potential benefits and harms of the intervention(s). | | | | | 5-6 |
|  | 3c**^a^** | Define which events are considered harms according to previous literature and provide a clear rationale for the specific harms included in the OoSRs. | | | | | NA |
| 4. Objectives  (PICOS) | 4 | Provide an explicit statement of research question(s) that specifies PICOS: | | | | | 6 |
|  |  | \| y \| \| --- \|  - Participants | \| y \| \| --- \|  - Interventions | \| y \| \| --- \|  - Comparators | \| y \| \| --- \|  - Outcomes | \| y \| \| --- \|  - Study design |  |
| **METHODS** | | | | | | |  |
| 5. Protocol and registration | 5a | Indicate clearly if a protocol exists or not. | | | | | 6 |
|  | 5b | If registered, provide the name of the registry (such as a valid Web address, PROSPERO). | | | | | 6 |
| 6. Eligibility criteria  & outcomes of interest | 6a | Specify inclusion and exclusion criteria for study design, participants, interventions and comparators in detail. | | | | | 6-8 |
|  | 6b | List (and define whenever it is necessary) the outcomes for which data were recorded, ideally include prioritization of main and additional outcomes. | | | | | 7-8 |
|  | 6c | Include adverse events as (primary or secondary) outcome of interest. Define them and grade their severity (such as mild, moderate, severe, fatal; severity could also be described in the appendix), if appropriate. | | | | | NA |
|  | 6d**^b^** | Specify report characteristics (such as language restrictions, publication status, and years considered) used as criteria for eligibility for the OoSRs (see also item 7). | | | | | 8 |
| 7. Information sources | 7a | Search at least two electronic bases. | | | | | 8 |
|  | 7b | Search supplementary sources (e.g. hand-searching, reference lists, related reviews and guidelines, protocol registries, conference abstracts, and other gray literature). | | | | | 8 |
|  | 7c | Report the date last searched and/or dates of coverage for each database. | | | | | 8 |
| 8. Search strategy**^c^** | 8a | Specify full electronic search strategy (algorithm) for at least one database including any limits used (e.g. language and date restrictions-see also subitems 6d and 7c) such that it could be repeated. | | | | | Appendix B |
|  | 8b | Present any additional search process (e.g. algorithm or filter for adverse events, searches in pertinent websites) specifically to identify adverse events that have been investigated. | | | | | NA |
| 9. Data management &  selection process | 9a**^d^** | Describe the software that was used to manage records and data throughout the OoSRs. | | | | | 8 |
|  | 9b | Define what is a SR and provide the process for selecting SRs and its relevant details (screening the title and abstract or full text by at least two reviewers, selection by multiple independent investigators and resolving disagreements by consensus). | | | | | 8 |
|  | 9c | Report any attempt to handle overlapping (include one review among multiple potential candidates by choosing for example the most updated SR, the most methodologically rigorous SR or the SR with larger number of primary studies). | | | | | NA |
| 10. Additional search for  primary studies | 10 | Report additional search to identify eligible primary studies (e.g. searching in more databases or update the search) and its relevant details. | | | | | NA |
| 11. Data collection process | 11a | Describe the method of data extraction from included SRs (e.g. data collection form, extraction in duplicate and independently, resolving disagreements by consensus). | | | | | 9 |
|  | 11b | Report any processes for obtaining, confirming or updating data from investigators (e.g. contact with authors of included reviews, obtain data from primary studies of included reviews). | | | | | 9 |
| 12. Data items | 12 | List (and define whenever is necessary) the specific variables for which data were recorded (e.g. PICOS items, number of included studies and participants, dose, length of follow up, results, funding sources) and any data assumptions and simplifications made. | | | | | 9 |
| 13. Assessment of methodological quality & quality of evidence | 13a | State the evaluation of reporting or/and methodological quality (eg. using PRISMA or PRISMA-harms, AMSTAR or R-AMSTAR) of the included reviews. | | | | | 9 |
|  | 13b**^e^** | State the evaluation of quality for individual studies that were included in the SRs (inform whether tools such as Jadad or RoB of Cochrane were used by the included reviews) and for the additional primary studies. | | | | | NA |
|  | 13c | State the evaluation of quality of evidence (e.g. using GRADE approach). | | | | | NA |
|  | 13d | Describe the methods (e.g. piloted forms, independently, in duplicate) used for the quality assessment. | | | | | 9-10 |
| 14. Meta-bias(es) | 14 | Specify any planned assessment of meta-bias(es) (such as publication bias or selective reporting across studies, ROBIS tool). | | | | | NA |
| 15. Data synthesis | 15a | Specify clearly the method (narrative, meta-analysis or network meta-analysis) of handling or synthesizing data and their details (e.g. state the principal summary measures that were extracted or calculated, how heterogeneity was assessed, what statistical approaches were used if a quantitative synthesis has been conducted). | | | | | 10 |
|  | 15b | Describe the software that was used to analyze the data if a quantitative synthesis has been conducted. | | | | | NA |
|  | 15c | Report if zero events are included in the studies and how they were handled in statistical analyses, if relevant. | | | | | NA |
|  | 15d | Describe methods of any pre-specified additional analyses (such as sensitivity or subgroup analyses, meta-regression). | | | | | NA |
| **RESULTS** | | | | | | |  |
| 16. Review & primary study selection | 16a | Provide the details of review selection (e.g. numbers of reviews screened, retrieved, and included and excluded in the overview) and the number of the additional eligible primary studies that were included, ideally with a flow diagram of the overview process. | | | | | 10 |
|  | 16b | Present a flow diagram that gives separately the number of studies focused on harms outcomes. | | | | | Figure 1 |
|  | 16c**^c^** | List the studies (full citation) that were excluded after reading the full text and provide reasons. | | | | | NA |
| 17. Review & primary study characteristics | 17a**^c^** | Describe characteristics of each included SR in tables (such as title or author, search date, PICOS, design and number of studies included, number and age range of participants, dose/frequency, follow up period [treatment duration], review limitations, results or conclusion) and of each additional primary study. | | | | | 11-12,  Tables 1-2 |
|  | 17b | For each included SR report language and publication status restrictions that have been used. | | | | | NA |
| 18. Overlapping | 18 | Present or/and discuss about overlapping of studies within SRs (at least one of the following): | | | | | 11,  Appendix C |
|  |  | - Present measures of overlap (such as CCA). | | | | | NA |
|  |  | - Provide citation matrix.**^c^** | | | | | Appendix C |
|  |  | - Give the number of index publications or/and discuss about overlapping.**^f^** | | | | | 11 |
| 19. Present assessment of methodological quality & quality of evidence | 19 | Present results in text or/and tables**^c^** of any quality assessment (see also subitems 13a-c): | | | | | 12,  Table 2,  Appendix D |
|  |  | - Reporting or/and methodological quality of the included SRs. | | | | | 12,  Table 2,  Appendix D |
|  |  | - Inform for the quality of the individual studies that were included in the SRs (report results for sequence generation, allocation concealment, blinding, withdrawals, bias etc.) and for the additional included primary studies. | | | | | NA |
|  |  | - Quality of evidence. | | | | | NA |
| 20. Present meta-bias(es) | 20 | Present results of any assessment of meta-bias(es) (such as publication bias or selective reporting across studies, ROBIS assessment). | | | | | NA |
| 21. Synthesis of results | 21a | Summarize and present the main findings of the overview for benefits and harms. If a quantitative synthesis has been conducted, present each summary measure with a confidence interval, prediction interval or a credible interval and measures of heterogeneity or inconsistency. | | | | | 13-23,  Tables 3-4,  Appendix E |
|  | 21b | Give results of any additional analyses (such as sensitivity, subgroup analyses, or meta-regression). | | | | | NA |
|  | 21c | Report results for adverse events separately for each intervention. | | | | | NA |
| **DISCUSSION** | | | | | | |  |
| 22. Summary of evidence | 22 | Provide a concise summary of the main findings with the strength and shortcomings of evidence for each main outcome. | | | | | 24-25 |
| 23. Limitations | 23a | Discuss limitations of either the overview or included studies (or both) (e.g. different eligibility criteria, limitations of searching reviews, language restrictions, publication and selection bias). | | | | | 25-26 |
|  | 23b | Report possible limitations of the included reviews related to harms (issues of missing data and information, definitions of harms, rare adverse effects). | | | | | NA |
| 24. Conclusions | 24a | Provide a general interpretation of the results in coherence with the review findings and present implications for practice; consider the harms equally as carefully as the benefits and in the context of other evidence. | | | | | 26 |
|  | 24b | Present implications for future research. | | | | | 26 |
| **AUTHORSHIP** | | | | | | |  |
| 25. Contributions of authors | 25 | Provide contributions of authors. | | | | | 38-39 |
| 26. Dual (co-)authorship | 26 | Report about dual (co-)authorship in the limitation or declarations of interest section. | | | | | 38-39 |
| **FUNDING** | | | | | | |  |
| 27. Funding or other support | 27a | Indicate sources of financial and other support for the OoSRs (direct funding) or for the authors (indirect funding), or report no funding. | | | | | 38 |
|  | 27b | Provide name for the overview funder and/or sponsor, or for the authors’ supporters. | | | | | 5, 38 |
|  | 27c | Describe roles of funder(s), sponsor(s), and/or institution(s), if any, in conducted the OoSRs. | | | | | 38-39 |

***Abbreviations:*** *OoSRs, Overview of Systematic Reviews; SRs, Systematic Reviews; PICOS, participants, interventions, comparisons, outcomes, and study design; CCA, corrected covered area.*

^a^Applicable mainly for OoSRs that focus on adverse events. The description could be placed in methods section. ^b^Language restrictions, publication status, and years could also be reported in information sources topic—see item 7. ^c^It could also be placed in an appendix as a supplementary material. ^d^The software used for the management of the records and data could be placed in the data collection process—see item 11. ^e^The way of evaluation (e.g. instruments) can be reported in item 19. ^f^Index publication is the first occurrence of a primary publication in the included reviews. Discussion for overlapping might be placed in the discussion section.

*Modified and extended for Overviews of Systematic Reviews (OoSRs) from:*  Moher D, Liberati A, Tetzlaff J, Altman DG, The PRISMA Group (2009). Preferred Reporting Items for Systematic Reviews and Meta-Analyses: The PRISMA Statement. PLoS Med 6(7): e1000097. <https://doi.org/10.1371/journal.pmed.1000097>

# Appendix B: Database Search Strategy

Database: Ovid MEDLINE: Epub Ahead of Print, In-Process & Other Non-Indexed Citations, Ovid MEDLINE® Daily and Ovid MEDLINE® <1946-Present>

Search Strategy:

--------------------------------------------------------------------------------

1 exp Emergency Responders/ (11395)

2 ((emergenc* or first) adj3 responder*).tw,kf. (2628)

3 ((frontline* or front line) adj3 responder?).tw,kf. (16)

4 (safety adj3 personnel).tw,kf. (472)

5 (firefighter* or fire fighter* or firem#n* or fire marshall?).tw,kf. (2514)

6 (medic or medics or paramedic? or para-medic?).tw,kf. (6226)

7 ((ambulance* or emergenc* or fire or correctional or corrections or prison? or rescue or "911" or "9-1-1") adj2 (employee? or officer? or personnel or staff or technician? or worker?)).tw,kf. (5259)

8 ((ambulance* or emergenc* or fire or correctional or rescue or "911" or "9-1-1") adj2 service?).tw,kf. (18762)

9 fire department?.tw,kf. (684)

10 (police or policeforce* or policeman* or policemen* or policewoman* or policewomen*).tw,kf. (13325)

11 (law enforcer? or law enforcement officer?).tw,kf. (419)

12 "Coroners and Medical Examiners"/ (1994)

13 (coroner? or medical examiner?).tw,kf. (5468)

14 (forensic adj2 (pathologist? or scientist?)).tw,kf. (1542)

15 or/1-14 [EMERGENCY SERVICES PERSONNEL] (56032)

16 px.fs. [psychology - floating subheading] (973323)

17 exp Adaptation, Psychological/ (120258)

18 Psychological Trauma/ (743)

19 Resilience, Psychological/ (4315)

20 resilien*.tw,kf. (25912)

21 Stress, Psychological/ (111899)

22 Stress Disorders, Post-Traumatic/ (29427)

23 (PTSD or PTSDs).tw,kf. (21704)

24 (stress or stressed or stresses or stressful* or stressing or distress*).tw,kf. (837701)

25 exp Anxiety Disorders/ (75534)

26 exp Depressive Disorder/ (102042)

27 Occupational Diseases/ (81560)

28 exp Occupational Stress/ (10920)

29 ((occupation* or job? or job-related or work or workplace or work-related) adj3 (disease* or ill or illness* or injur* or sick*)).tw,kf. (27069)

30 exp Suicide/ (58433)

31 (suicid* or parasuicid* or para-suicid*).tw,kf. (71419)

32 exp Substance-Related Disorders/ (263589)

33 (pharmacotherap* or pharmaco-therap*).tw,kf. (33090)

34 (pharmaceutical* adj2 (interven* or treat* or therapy or therapies)).tw,kf. (3592)

35 (addict* adj3 (prevent* or rehabil* or treat* or therapy or therapies)).tw,kf. (8634)

36 ((substance? or drug?) adj2 (abus* or misus* or "use" or used or user? or using) adj5 (prevent* or rehabil* or treat* or therapy or therapies)).tw,kf. (32950)

37 burnout?.tw,kf. (9064)

38 ((compassion* or empath*) adj3 fatigue).tw,kf. (607)

39 ((secondary or vicarious*) adj3 trauma*).tw,kf. (4094)

40 Mental Health/ (32863)

41 (cope or coped or copes or coping).tw,kf. (75977)

42 ("mental health" or "mental well-being" or "mental wellbeing" or "emotional health" or "emotional well-being" or "emotional wellbeing" or "psychological health" or "psychological well-being" or "psychological wellbeing" or "social well-being" or "social wellbeing" or (employee* adj2 wellness*) or (employee* adj2 wellbeing) or (employee* adj2 well-being) or (staff adj2 wellness*) or (staff adj2 wellbeing) or (staff adj2 well-being) or (workplace* adj2 wellness*) or (workplace* adj2 wellbeing) or (workplace* adj2 well-being) or (work place* adj2 wellness*) or (work place* adj2 wellbeing) or (work place* adj2 well-being)).tw,kf. (150344)

43 ((employee* adj2 health*) or (staff adj2 health*) or (workplace* adj2 health*) or (work place* adj2 health*)).tw,kf. (11497)

44 Health Promotion/ (68547)

45 ((health or wellbeing or well-being or wellness) adj3 (campaign* or promot*)).tw,kf. (56114)

46 (program* adj3 access*).tw,kf. (4313)

47 ((posttrauma* or post-trauma*) adj3 grow$3).tw,kf. (1223)

48 survivorship.tw,kf. (11795)

49 ((behav* or cognitive* or emotion* or psycholog*) adj3 (adapt* or adjust* or change* or changing or grew or grow or grows or growth* or orient* or rehabil*)).tw,kf. (105443)

50 (positiv* adj3 (adapt* or adjust* or change* or changing or grew or grow or grows or growth* or orient* or rehabil*)).tw,kf. (24500)

51 ((emotion* or mental* or psychological*) adj3 (heal or healed or healing or heals or recover* or rehabil*)).tw,kf. (4352)

52 ((post-disaster? or post-event? or post-incident? or posttrauma* or post-trauma*) adj3 (heal or healed or healing or heals or recover* or rehabil*)).tw,kf. (506)

53 Crisis Intervention/ (5535)

54 (cris#s adj3 (respond* or respons* or interven*)).tw,kf. (2673)

55 Hotlines/ (2605)

56 hotline?.tw,kf. (1119)

57 Behavioral Risk Factor Surveillance System/ (1828)

58 BRFSS.tw,kf. (1372)

59 (risk? adj3 (monitor* or survey or surveillance* or watch*)).tw,kf. (13574)

60 Risk Taking/ (25378)

61 ((take or takes or taking or took) adj2 risk?).tw,kf. (8391)

62 (behav* adj2 risk?).tw,kf. (35945)

63 Organizational Culture/ (16374)

64 ((organi#ation* or corporate or corporation* or job or jobs or job-related or professional* or safety or work or workplace or work-related) adj3 (climate? or cultur* or value or valued or values or valuing or belief? or believ*)).tw,kf. (21319)

65 Leadership/ (38258)

66 (leader or leaders or leadership).tw,kf. (68882)

67 (influencer? or influential?).tw,kf. (19145)

68 ((organi#ation* or corporate or corporation* or job or jobs or job-related or professional* or safety or work or workplace or work-related) adj3 (leader* or manager* or management*)).tw,kf. (18271)

69 exp Social Support/ (66056)

70 ((social* or peer? or psychoeducat* or psycho-educat* or psychological* or psychosocial* or psycho-social*) adj3 (interven* or rehabil* or support* or treat* or therapy or therapies)).tw,kf. (87132)

71 ((digital* or online or internet or internet-based or text-messag* or web or web-based) adj3 (interven* or rehabil* or support* or treat* or therapy or therapies)).tw,kf. (12055)

72 exp Psychotherapy/ (184601)

73 (psychotherap* or psycho-therap*).tw,kf. (43871)

74 ((organi#ation* or corporate or corporation* or job or jobs or job-related or professional* or safety or work or workplace or work-related) adj3 support*).tw,kf. (24858)

75 exp Self-Help Groups/ (9815)

76 ((assess* or help*) adj2 (personal* or self or selves or himself or herself or themsel*)).tw,kf. (34698)

77 Social Stigma/ (6168)

78 (stigma* or shame* or embarrass*).tw,kf. (39728)

79 Absenteeism/ (8644)

80 absentee*.tw,kf. (5608)

81 Presenteeism/ (192)

82 presentee*.tw,kw. (1113)

83 ((work* or employ*) adj5 (absenc* or absent* or presenc* or present*)).tw,kw. (109177)

84 ((work* or employ*) adj5 abilit*).tw,kf. (10479)

85 (time? adj1 away).tw,kw. (609)

86 Sick Leave/ (5396)

87 ((sick or medical) adj leave?).tw,kw. (4899)

88 ((missed or lost) adj2 work*).tw,kw. (2748)

89 or/16-88 [STRESS/RESILIENCE/ETC.] (2911922)

90 15 and 89 [EMERGENCY SERVICES PERSONNEL - STRESS/RESILIENCE/ETC.] (17123)

91 limit 90 to "systematic reviews" (450)

92 systematic review.pt. (101912)

93 meta analysis.pt. (97298)

94 exp meta-analysis as topic/ (17375)

95 (meta-analy* or metanaly* or metaanaly* or met analy* or integrative research or integrative review* or integrative overview* or research integration or research overview* or collaborative review*).tw,kf. (146901)

96 (systematic review* or systematic overview* or evidence-based review* or evidence-based overview* or (evidence adj3 (review* or overview*)) or meta-review* or meta-overview* or meta-synthes* or rapid review* or "review of reviews" or umbrella review? or technology assessment* or HTA or HTAs).tw,kf. (181367)

97 exp Technology assessment, biomedical/ (10615)

98 (cochrane or health technology assessment or evidence report or systematic reviews).jw. (18122)

99 (network adj (MA or MAs)).tw,kf. (5)

100 (NMA or NMAs or MTC or MTCs or MAIC or MAICs).tw,kf. (6249)

101 indirect* compar*.tw,kf. (1649)

102 (indirect treatment* adj1 compar*).tw,kf. (197)

103 (mixed treatment* adj1 compar*).tw,kf. (458)

104 (multiple treatment* adj1 compar*).tw,kf. (148)

105 (multi-treatment* adj1 compar*).tw,kf. (0)

106 simultaneous* compar*.tw,kf. (984)

107 mixed comparison?.tw,kf. (24)

108 or/92-107 (313926)

109 90 and 108 (279)

110 91 or 109 [REVIEWS/MAs] (489)

111 exp Animals/ not Humans/ (4548295)

112 110 not 111 [ANIMAL-ONLY REMOVED] (488)

113 limit 112 to yr="2009-current" (347)

114 limit 113 to english (339)

# Appendix C: Overlap table

| **Study Author**  **(Year)** | **Review Author (Year)** | | | | | | | | | | | | | | **Times Cited** | **Population** |
| --- | --- | --- | --- | --- | --- | --- | --- | --- | --- | --- | --- | --- | --- | --- | --- | --- |
|  | Patterson (2014) | Rose (2016) | Haugen (2012) | MacMillan (2017) | Lees (2019) | Barger (2018) | Witt (2017) | Skeffington (2013) | Torchalla (2018) | Whybrow (2015) | Plat (2011) | Milner (2014) | Stergiopoulos (2011) | Martin (2009) |  |  |
| Gersons  (2000) | X |  | X |  |  |  |  |  | X |  |  |  | X |  | 4 | Police |
| Arnetz  (2009) |  |  |  |  |  |  |  | X |  |  | X |  |  |  | 2 | Police |
| Christopher  (2016) |  |  |  |  | X | X |  |  |  |  |  |  |  |  | 2 | Police |
| Kuehl  (2016) |  |  |  | X |  | X |  |  |  |  |  |  |  |  | 2 | Police |
| Lansing  (2005) |  |  | X |  |  |  |  |  | X |  |  |  |  |  | 2 | Police |
| Mishara  (2012) |  |  |  |  |  |  | X |  |  |  |  | X |  |  | 2 | Police |
| Norvell  (1993) | X |  |  | X |  |  |  |  |  |  |  |  |  |  | 2 | Police |
| Richmond  (1999) | X |  |  | X |  |  |  |  |  |  |  |  |  |  | 2 | Police |
| Short  (1984) | X |  |  | X |  |  |  |  |  |  |  |  |  |  | 2 | Police |
| Tanigoshi  (2008) | X |  |  | X |  |  |  |  |  |  |  |  |  |  | 2 | Police |
| Wilson  (2001) | X |  | X |  |  |  |  |  |  |  |  |  |  |  | 2 | Police |
| Ackerley  (1986) | X |  |  |  |  |  |  |  |  |  |  |  |  |  | 1 | Police |
| Arble  (2017) |  |  |  |  | X |  |  |  |  |  |  |  |  |  | 1 | Police |
| Bohl  (1991) |  | X |  |  |  |  |  |  |  |  |  |  |  |  | 1 | Police |
| Carlier  (2000) |  | X |  |  |  |  |  |  |  |  |  |  |  |  | 1 | Police |
| Chongruksa  (2012) |  |  |  |  | X |  |  |  |  |  |  |  |  |  | 1 | Police |
| Cornelius  (2007) |  |  | X |  |  |  |  |  |  |  |  |  |  |  | 1 | Police |
| Coulson  (1987) | X |  |  |  |  |  |  |  |  |  |  |  |  |  | 1 | Police |
| Digliani  (1994) | X |  |  |  |  |  |  |  |  |  |  |  |  |  | 1 | Police |
| Ford  (1996) |  |  | X |  |  |  |  |  |  |  |  |  |  |  | 1 | Police |
| Holbrook  (1994) |  |  |  |  |  | X |  |  |  |  |  |  |  |  | 1 | Police |
| Hunt  (2013) |  |  |  |  |  |  |  |  |  | X |  |  |  |  | 1 | Police |
| Ireland  (2007) | X |  |  |  |  |  |  |  |  |  |  |  |  |  | 1 | Police |
| Jeter  (2013) |  |  |  | X |  |  |  |  |  |  |  |  |  |  | 1 | Police |
| Leonard  (1999) |  | X |  |  |  |  |  |  |  |  |  |  |  |  | 1 | Police |
| Levenson  (2010) |  |  |  |  |  |  | X |  |  |  |  |  |  |  | 1 | Police |
| McCraty  (1999) | X |  |  |  |  |  |  |  |  |  |  |  |  |  | 1 | Police |
| Norris  (1990) |  |  |  | X |  |  |  |  |  |  |  |  |  |  | 1 | Police |
| Oliver  (2009) |  |  |  |  | X |  |  |  |  |  |  |  |  |  | 1 | Police |
| Peres  (2011) |  |  |  |  | X |  |  |  |  |  |  |  |  |  | 1 | Police |
| Plat  (2013) |  |  |  |  | X |  |  |  |  |  |  |  |  |  | 1 | Police |
| Sarason  (1979) |  |  |  |  |  |  |  | X |  |  |  |  |  |  | 1 | Police |
| Shipley  (2002) | X |  |  |  |  |  |  |  |  |  |  |  |  |  | 1 | Police |
| Sijaric-Voloder  (2008) |  |  |  |  |  |  |  | X |  |  |  |  |  |  | 1 | Police |
| Tolin  (1999) |  |  | X |  |  |  |  |  |  |  |  |  |  |  | 1 | Police |
| Watson  (unpublished, 2014) |  |  |  |  |  |  |  |  |  | X |  |  |  |  | 1 | Police |
| Welch  (1998) |  |  |  |  |  |  | X |  |  |  |  |  |  |  | 1 | Police |
| Bohl  (1995) |  | X |  |  |  |  |  |  |  |  |  |  |  |  | 1 | Firefighters |
| Coupland  (2009) |  |  | X |  |  |  |  |  |  |  |  |  |  |  | 1 | Firefighters |
| Finney  (2015) |  |  |  |  |  |  | X |  |  |  |  |  |  |  | 1 | Firefighters |
| Harris  (2002) |  | X |  |  |  |  |  |  |  |  |  |  |  |  | 1 | Firefighters |
| Kitchinier  (2004) |  |  | X |  |  |  |  |  |  |  |  |  |  |  | 1 | Firefighters |
| Regehr  (2001) |  | X |  |  |  |  |  |  |  |  |  |  |  |  | 1 | Firefighters |
| Sullivan  (2017) |  |  |  |  |  | X |  |  |  |  |  |  |  |  | 1 | Firefighters |
| Tuckey  (2014) |  | X |  |  |  |  |  |  |  |  |  |  |  |  | 1 | Firefighters |
| McCraty  (2003) |  |  |  |  |  |  |  |  |  |  |  |  |  | X | 1 | Correctional |
| Ruck  (2013) |  | X |  |  |  |  |  |  |  |  |  |  |  |  | 1 | Correctional |
| **Studies Cited** | 12 | 8 | 8 | 7 | 6 | 4 | 4 | 3 | 2 | 2 | 2 | 1 | 1 | 1 |  |  |

# Appendix D: Quality appraisal of included reviews (AMSTAR-2 Results)

| **Author**  **(Year)** | 1. PICO components | 2. A priori design* | 3. Rationale for study selection | 4. Literature search* | 5. Duplicate Selection | 6. Duplicate Abstraction | 7. List of excluded studies* | 8. Description of included studies | 9a. Risk of Bias assessment in RCTs* | 9b. Risk of bias assessment in non-randomised studies* | 10. Funding sources | 11. Appropriate MA methods* | 12. Used RoB in MA | 13. Used RoB in interpreting results* | 14. Discussion of heterogeneity | 15. Publication bias* | 16. Conflict of Interest | **Overall Rating** |
| --- | --- | --- | --- | --- | --- | --- | --- | --- | --- | --- | --- | --- | --- | --- | --- | --- | --- | --- |
| Lees, T (2019) | Y | N | N | P | Y | N | N | N | Includes only NRCTs | Y | N | No MA | No MA | N | No MA | No MA | N | **Critically Low** |
| Barger, LK (2018) | Y | Y | N | P | Y | Y | Y | Y | Y | Y | N | Y | N | Y | Y | N | N | **Moderate** |
| Patterson, PD (2018) | Y | Y | N | P | Y | Y | Y | Y | Y | Y | N | No MA | No MA | Y | No MA | No MA | N | **High** |
| Torchalla, I (2018) | Y | N | Y | P | Y | Y | N | Y | Y | Y | N | Y | Y | Y | Y | N | Y | **Critically Low** |
| MacMillan, F (2017) | Y | N | N | P | Y | Y | N | Y | Includes only NRCTs | Y | N | No MA | No MA | Y | No MA | No MA | Y | **Low** |
| Witt, K  (2017) | Y | N | N | P | Y | Y | N | Y | N | N | N | Y | N | Y | N | N | Y | **Critically Low** |
| Rose, FR  (2016) | Y | Y | Y | Y | N | N | N | Y | N | N | N | N | No | N | N | N | N | **Critically Low** |
| Whybrow, D (2015) | Y | N | N | P | N | N | N | PY | N | N | N | No MA | No MA | N | No MA | No MA | Y | **Critically Low** |
| Milner, A  (2014) | Y | N | N | P | N | N | Y | Y | Includes only NRCTs | N | N | No MA | No MA | N | No MA | No MA | N | **Critically Low** |
| Skeffington, PM (2013) | Y | N | N | P | N | N | N | Y | Includes only NRCTs | N | N | No MA | No MA | N | No MA | No MA | N | **Critically Low** |
| Haugen, PT (2012) | Y | N | N | P | N | N | N | Y | N | N | N | No MA | No MA | N | No MA | No MA | N | **Critically Low** |
| Plat, MJ  (2011) | Y | N | N | P | N | N | N | Y | Y | Y | N | No MA | No MA | Y | No MA | No MA | Y | **Low** |
| Stergiopoulos, E (2011) | Y | P | N | P | Y | N | N | Y | P | Y | N | No MA | No MA | Y | No MA | No MA | Y | **Moderate** |
| Martin, A  (2009) | Y | N | N | P | Y | Y | N | P | N | N | N | Y | N | N | Y | N | N | **Critically Low** |

**Abbreviations**: **MA** – Meta-analysis; **N** – No; **NA** – Not applicable; **NRCT** – Non-randomised controlled trial; **P** – Partial yes; **RCT** ­– Randomised controlled trial; **Y** – Yes

**Notes**: *These were considered critical domains and were subsequently given greater weight in the overall AMSTAR 2 rating.

# Appendix E: Details of included primary studies

## Table E1. Police studies

| **Study author (year)** | **Study country** | **Study objective(s)** | **Methods** | **Results** | **Conclusion** |
| --- | --- | --- | --- | --- | --- |
| Arble, E (2017) | USA | This study aimed to conduct a preliminary test of the imagery-based trauma prevention program to improve coping and prevent mental health problems among new police officers during their first year working in a high-threat urban environment. | In an uncontrolled demonstration project, junior officers were trained by senior officers to engage in imaginal rehearsal of specific dangerous situations while incorporating optimal police tactics and healthy emotional reactions. A class of 32 officers in the police academy engaged in the program, and they and the trainers reported high satisfaction with it. After their first year of field work, 22 officers were reassessed. | Compared to pre-training, these officers showed significant increases in the use of positive reframing and humor and significant reductions in anxiety and alcohol use over the year. | These results offer preliminary evidence for the feasibility and effectiveness of this trauma prevention program for new police officers. |
| Christopher, MS (2016) | USA | This pilot study examined the feasibility and preliminary effectiveness of a mindfulness-based intervention designed to address police officer stress. | A total of 43 police officers completed an 8-week Mindfulness-Based Resilience Training (MBRT) program, which was designed to improve mindfulness, resilience, stress, health outcomes, and emotional functioning. | Using multilevel models, the study found significant improvement in self-reported mindfulness, resilience, police and perceived stress, burnout, emotional intelligence, difficulties with emotion regulation, mental health, physical health, anger, fatigue, and sleep disturbance. Although there were no significant pre-to-post-MBRT changes in cortisol awakening response (CAR), while controlling for pre-MBRT increase area under the curve (AUCI), change in mental health was a significant predictor of post-AUCI. | Given voluminous research on stress and related consequences, and the dearth of research on effective stress management interventions among police officers, these preliminary findings suggest that an 8-week intervention can be both feasible and effective. Continued exploration in this area may help us understand and measure mental qualities that lead to improvements in health and functioning observed in MBRT. |
| Kuehl, KS (2016) | USA | The SHIELD (Safety & Health Improvement: Enhancing Law Enforcement Departments) Study is a worksite wellness team-based intervention among police and sheriff departments which aimed to assess the program’s effectiveness to reduce occupational risks and unhealthy lifestyle behaviors. | Both intervention and control groups were followed for 24 months, and we report those durability findings, along with qualitative group interview results that provide insight into the changes of the long-term outcomes. | The SHIELD team-based health promotion program was found to be feasible and effective at 6 months in improving diet, sleep, stress, and overall quality of life of law enforcement department personnel. Long-term effects were observed for consumption of fruits and vegetables, and there was some evidence for effects on tobacco and alcohol use. | The SHIELD program was feasible, effective, and durable for improving dietary changes. |
| Watson (2014)^†^ | UK | This study aimed to examine the association of the Trauma Risk Management (TRiM) program with stigma and attitudes to stress and trauma among police officers. | This cross-sectional study of 859 operational and non-operational police officers compared self-reported psychological distress, attitudes towards help seeking, stigma and post-traumatic psychological growth among those who attended TRiM sessions and those who did not. A total of 693 police officers participated in the TRiM program, with 166 not participating in any post-trauma risk management program. | Those in the TRiM group reported significantly lower stigma, lower psychological distress, greater positive post-traumatic change and fewer barriers to help seeking, than those in the non-TRiM group. However, there was no significant difference between the groups in terms of attitudes towards stress and PTSD. | The authors conclude that even in forces using TRiM, there is still a considerable degree of stigma, particularly public- stigma, and barriers to seeking help for psychological distress, which needs to be addressed in future research and development of interventions. This research does, however, offer preliminary support for the use of TRiM within police settings. |
| Hunt, E (2013) | UK | The Cumbrian Constabulary deployed an organizational peer support response for personnel involved known as TRiM. The aim was to examine data routinely gathered during the TRiM process to evaluate the relationship of the intervention to sickness absence. | Using incident databases, details were gathered regarding exposure to the murders and type of TRiM intervention, including an assessment of the psychological risk to the individual of developing a trauma-related mental health problem. Sociodemographic information was collated by the occupational health department. Cumulative sickness absence data in the 2 months following the murders were used as a proxy for mental health status. | A total of 717 police officers and civilian support staff were identified. High levels of traumatic exposure were associated with subsequent receipt of a TRiM intervention. The majority of psychological risk indices reduced between the initial and subsequent evaluation. Greater traumatic exposure was associated with longer sickness absence lengths. Engagement in the TRiM process was associated with a reduction in sickness absence especially in more junior ranks. | The study found that TRiM deployed within a police force responding to a major event offered a way of structuring a response for those involved. The data suggests that TRiM may offer a way of assessing psychological risk so that officers can be offered early supportive interventions, and suggests that TRiM may help to ameliorate some of the negative effects of high trauma exposure. |
| Jeter, PE (2013) | USA | This pilot study evaluated the effects of Kripalu yoga on perceived stress, mood, and mindfulness during police academy training. | Forty-two recruits participated in a 6-class yoga intervention. Participants completed the Profile of Mood States-Short Form, Perceived Stress Scale, and the Five Facet Mindfulness Questionnaire prior to and immediately following completion of the yoga program, as well as an exit survey. | Paired samples t-tests revealed significant post-intervention changes in perceived stress and mood, reductions in tension and fatigue, and a trend toward reduced anger. Changes in mindfulness were not detected. The exit survey indicated perceived benefits of yoga for some participants. | This preliminary study suggests that yoga may be beneficial for reducing stress, tension, and fatigue among police academy trainees. Future longitudinal randomised controlled trials are needed to evaluate its full potential as a permanent component of police academy training. |
| Plat, MCJ (2013) | NR | This study i) describes the number of police personnel with PTSD who are working and those who are on sick leave before and after an out-patient-clinic treatment program and ii) examines which factors are related to return to work. | Police personnel treated for PTSD (n = 121). In this retrospective study all police officers had an intake interview before and an outtake interview following a 16-week treatment for PTSD. Information about several personal characteristics, PTSD complaints, and work related factors were gathered. A t-test and chi-square test were used to evaluate differences between working police personnel and police personnel on sick leave at intake and outtake. Binary logistic regression was used to test whether the intake data were related to returning to work at outtake. | At the start of the treatment half of the police personnel were on sick leave (n = 59) and at outtake 48 participants who were not working at intake had returned to work. None of the variables at intake contributed significantly to return to work at outtake. | The majority of police officers returned to work after the treatment program. The study recommends that attention be paid to successful return to work as part of the treatment program, therefore the occupational health professional and employer should be involved. |
| Chongruksa, D (2012) | Thailand | This study aims to develop the eclectic group counseling intervention for Thai police officers to reduce the risk of developing symptoms of poor mental health while deploying in terrorist situations. | Our eclectic group counseling included the interactive model of Cognitive Behavioral Therapy, religious interventions, mandala drawing, and Reality Therapy. The design is an experiment-controlled trial with 42 participants (The experiment = 20, the control = 22; Thai-Buddhists = 32, Thai-Muslims = 10). | For participants attending the eclectic counseling group, all three effects showed a curvilinear trend with the drop of mental health symptoms at mid-intervention, after termination, and weakening at one-month follow up. In contrast, most effects for participants receiving the control showed more of a linear pattern of mental health symptom at mid-intervention, after termination, and at one-month follow up. | This study should be viewed as an initial step toward examining the longitudinal effects of eclectic group counseling on preventing symptoms of poor mental health. The addition of effect size at mid-intervention, termination and one-month follow up, aided in determining the degree to which initial positive outcomes were sustained over time. |
| Mishara, BL (2012) | Canada | The aim was to evaluate Together for Life, a suicide prevention program for the Montreal police. | All 4178 members of the Montreal police participated. The program involved training for all officers, supervisors, and union representatives as well as establishing a volunteer helpline and a publicity campaign. Outcome measures included suicide rates, pre-post assessments of learning, focus groups, interviews, and follow-up of supervisors. | In the 12 years since the program began the suicide rate decreased by 79% (6.4/100,000), while other Quebec police rates had a non-significant (11%) increase (29.0/100,000). Also, knowledge increased, supervisors engaged in effective interventions, and the activities were highly appreciated. | The decrease in suicides appears to be related to this program since suicide rates for comparable populations did not decrease and there were no major changes in functioning, training, or recruitment to explain the differences. Comprehensive suicide prevention programs tailored to the work environment may significantly impact suicide rates. |
| Peres, JFP (2011) | NR | The study investigated the brain activity underlying trauma, the coping effect of psychotherapy, and resilience in a highly homogenous sample that experienced the same traumatic event. | The design applied was a between-group comparison of cerebral blood-oxygenation-level-dependent signals and symptom scores of police officers with and without partial PTSD (pPTSD). We used functional magnetic resonance imaging (fMRI) to investigate the retrieval of traumatic memories of 36 volunteers divided in three groups: (1) pPTSD policemen submitted to psychotherapy; (2) pPTSD policemen on the wait list; and (3) symptom-free (resilient) policemen. All participants were given a baseline fMRI scan and a follow-up scan some 40 days later. Not given psychotherapy, groups 2 and 3 were controls. | Group 1 showed 37% fewer PTSD symptoms post-psychotherapy and their scores and neural expressions were comparable to Group 3 resilient policemen. A marked increase in medial prefrontal cortex (mPFC) activity was concomitant with decreased amygdala activity during traumatic memory retrieval in both resilient and pPTSD participants (after psychotherapy) and these findings were associated with symptom attenuation. | The results provide neurophysiological evidence of resilience in a high-risk group for PTSD. Psychotherapy may help to build narratives and resilient integrated translations of fragmented traumatic memories via mPFC activity, and thus weaken their sensory content while strengthening them cognitively. |
| Levenson, RL Jr (2010) | USA | This article discusses the Badge of Life Psychological Survival for Police Officers Program (BOL), founded in 2008, with the goal of developing an effective police officer suicide primary and secondary prevention program. | Along with standard suicide prevention protocols typically used in other programs, an Emotional Self Care Program (ESC) was designed to focus on the officer stability and responsibility to care for his own emotional well-being. The model relies on teaching the factor of resilience as a significant component of stress-resistance. Selected for their credibility and trust level, peer support officers conduct the actual annual training workshops, set the example, and encourage involvement at all levels. A "cradle-to-the-grave" program (i.e., rookies to retirees), | BOL began long-term efforts to bridge the gap by demonstrating not only their wealth of value in mental health programs, but by providing a structured means by which departments can feel more comfortable taking advantage of these rich-in-experience resources. Since its formation, BOL has grown with alacrity. BOL's membership is represented by police administrators, "road cops," officers who suffer from PTSD, and parents and wives who have lost their loved ones to suicide. | BOL continues to be a strong, innovative voice in the formulation of new ideas to improve the emotional well-being of law enforcement officers throughout the United States and Canada. Training and education in police officer suicide prevention remains the core of BOL, while parallel issues currently under study are the recognition and acceptance of what we term the Line of Duty Suicide (LODS) by police administrators and police officers, in general. |
| Arnetz, B  (2009) | Sweden | The objective of this study is to test the effects of police trauma resilience training on stress and performance during a critical incident police work simulation. | Rookie police officers (N=18) participated in a randomised trial of a 10-week imagery and skills training program versus training as usual. Twelve months later, psychophysiological stress and police work performance were assessed during a live critical incident simulation. | Training resulted in significantly less negative mood, less heart rate reactivity, a larger increase in antithrombin, and better police performance compared to controls. Trends for cortisol and self-reported stress also suggested benefits of training. | This novel training program is a promising paradigm for improving police well-being, stress resiliency, and optimizing job performance. |
| Oliver, WM (2009) | NR | This study assessed the impact that a stress management program had on small-town and rural police officers and sheriffs’ deputies in West Virginia. | Assessing the impact of stress management training on officers’ anxiety, self-reported levels of stress, and behaviors in a paired sample (pre-test/post-test) t-test. | Findings indicate that only one of the three measures employed, the Self-Report measure, was significant. In this case, the mean improved from 3.469 to 2.212. In the case of both the anxiety and behavioral scales, the means actually increased, but again, neither was found to be statistically significant. | Findings suggested that these programs do have an impact on officer stress, but as the lag time increases, there is a diminished return of benefit, supporting the need for continued education. |
| Sijaric-Voloder, S (2008) | Bosnia and Herzegovina | Objective was to develop a structured stress management program based on cognitive behavior therapy principles for persons in high risk professions such as police officers. Another objective was to evaluate the effects of structured stress management program on police officers by measuring their anxiety levels before and after the treatment. | The subjects were police officers, aged from 30 to 45 years, divided into two groups, each consisting of 20 persons. One group received structured stress management program and another did not. All members of both groups completed the Beck Anxiety Inventory (BAI) and Anxiety Sensitivity Index (ASI) tests ahead and after the treatment, as well as three months after the treatment. | Statistical data analyses revealed that the subjects who had received stress management program displayed a statistically significant anxiety sensitivity reduction, developed more active and planned coping strategies for stressful situations, had less somatic reactions to stress and have thus also achieved improved performance at work and in private life. The subjects were also significantly more likely to request medical advice after completing psychotherapeutic program. |  |
| Tanigoshi, H (2008) | Canada | The purpose of this exploratory study was to determine the effectiveness of wellness counseling on increasing wellness among law enforcement officers. The study also examined whether position in the Transtheoretical Model and self-efficacy influenced the effectiveness of counseling on wellness. | Five, 60-min cognitive-behavioural counseling, personalized wellness sessions were implemented every other week. Referral to mental health services as required. Outcomes were measured using Wellness (5F-Wel Survey), Self-efficacy (single-item self-efficacy scale), Readiness to change wellness (stage of Readiness to Change Questionnaire) scales. | The treatment of wellness counseling had a significant effect on increasing overall total wellness levels between pre-test (M = 217, SD = 23.44) and post-test (M = 234, SD = 22.67) measurements. The control group's wellness levels remained unchanged from pre-test to post-test. | These findings indicate that counseling may increase wellness among law enforcement officers. |
| Cornelius, TL (2007) | USA | The aim of this study was to identify the conceptual framework for treatment, explore the course of therapy, and identify socially valid means of treatment and assessment in outpatient therapy. | This case report addresses treatment of PTSD with a 72-year-old former police officer who was experiencing intrusive thoughts, nightmares, experiential and affective avoidance, and hyperarousal resulting from witnessing a series of traumatic events during his time in law enforcement. | Although the latency between events and treatment was more than 20 years, this patient was responsive to a cognitive-behavioral, exposure-based treatment. Several other factors, including the client’s age and chronic pain, complicated this case. | At the termination of treatment, this patient evidenced improvements in overall anxiety, depressive symptoms, and frequency and severity of PTSD symptoms. Recommendations for clinicians and treatment implications are also presented. |
| Ireland, M (2007) | Australia | This study examined the impact that writing about personal emotions had on distress levels in police officers. | A total of 67 police officers randomly assigned to either a writing intervention or a non-writing control group completed the study. Over a period of 4 work days the intervention group members wrote for 15 minutes a day about their strong emotions related to work or not, and what they planned to do about the emotions. All participants completed measures of stress, anxiety, and depression before and after the intervention. | The results showed that the writing group experienced significantly lower levels of stress and anxiety post-intervention than the control group. | The results suggest that writing about emotions can help reduce distress in police officers. |
| Lansing, K (2005) | USA | In this study, the authors evaluated the effectiveness and physiological effects of EMDR in police officers involved with on-duty shootings and who had PTSD. | Six police officers involved with on-duty shootings and subsequent delayed-onset PTSD were evaluated with standard measures, the Post-traumatic Stress Diagnostic Scale (PDS), and high-resolution brain single photon emission computed tomography (SPECT) imaging before and after treatment. | All police officers showed clinical improvement and marked reductions in the PDS. In addition, there were decreases in the left and right occipital lobe, left parietal lobe, and right pre-central frontal lobe as well as significantly increased perfusion in the left inferior frontal gyrus. | EMDR was an effective treatment for PTSD in this police officer group, showing both clinical and brain imaging changes. |
| Shipley, P (2002) | Canada | This study examined the effectiveness of visuo-motor behavior rehearsal (VMBR) as a method of reducing acute stress and improving police officer performance. | Fifty-four recruits were randomly assigned to a treatment and a non-treatment condition prior to undergoing a highly stressful, critical event training scenario involving “live fire.” | A manipulation check showed that participants who received VMBR displayed significantly lower scores on the cognitive state anxiety subscale of the Competitive State Anxiety Inventory-2; somatic state anxiety and self-confidence results were unaffected by the VMBR treatment. Most importantly, participants in the VMBR training condition displayed better performance on the critical event scenario, including significantly more assailant “hits.” | The findings of this study revealed that VMBR training can reduce cognitive state anxiety and improve police officer performance in a dynamic and stressful live-fire training simulation. The link between VMBR training and human performance under stress can be conceptualized in the context of a serial four-stage model of stress and performance. |
| Wilson, SA (2001) | USA | The present study investigates the use of EMDR in the field of police psychology, striking new ground in applying the treatment to an area of occupational stress management that has traditionally employed an educational approach. | Sixty-two police officers were randomly assigned to either EMDR or a standard stress management program (SMP), each consisting of 6 hours of individualized contact. | At completion, officers in the EMDR condition provided lower ratings on measures of PTSD symptoms, subjective distress, job stress, and anger; and higher marital satisfaction ratings than those in SMP. | The effects of EMDR were maintained at the 6-month follow-up, indicating enduring gains from a relatively brief treatment regimen for this subclinical sample of officers who were experiencing some level of stress from their job. |
| Carlier, IVE (2000) | Netherlands | The present study tests the hypothesis that debriefing reduces the psychological morbidity caused by work-related incidents. | As debriefing techniques were not designed for application on a ‘one-off’ basis, the procedure studied here consisted of three successive debriefing sessions (at 24 hours, 1 month and 3 months post-trauma), which included traumatic stress education. In a sample of 243 traumatized police officers, a subgroup of debriefed officers (N = 86) was compared with non-debriefed internal (N = 82) and external (N = 75) control groups. | No differences in psychological morbidity were found between the groups at pre-test, at 24 hours or at 6 months post-trauma. One week post-trauma, debriefed subjects exhibited significantly more post-traumatic stress disorder symptomatology than non-debriefed subjects. High levels of satisfaction with debriefing were not reflected in positive outcomes. | Study findings on satisfaction are in agreement with evidence that participants generally appreciate the intervention. This seems to reflect a natural tendency in people who have recently been traumatized to seek emotional support, recognition, understanding and endorsement. Nevertheless, this high degree of satisfaction with debriefing was in no way reflected in fewer stress symptoms, lower rates of sick leave or a more rapid resumption of work. In the future, studies of the efficacy of group, as opposed to individual, debriefing are needed. |
| Gersons, BPR (2000) | The Netherlands | The present study investigated the combined effect of the cognitive–behavioral and psychodynamic approaches within one and the same treatment method. | Forty-two police officers with the diagnosis of PTSD participated in the study; 22 were randomly assigned to the treatment group and 20 to the wait-list control group. Assessments of PTSD and comorbid conditions were made 1 week before treatment, after treatment session 4, upon termination of treatment (16 sessions), and at follow-up 3 months later. | As expected, no significant differences between groups were observed at pre-test or at session 4. At posttest and at follow-up, BEP had produced significant improvement in PTSD, in work resumption, and in some comorbid conditions. | BEP treatment was effective for all three PTSD symptom clusters and showed clinically significant change. |
| Leonard, R (1999) | Australia | This study investigates appraisal and coping behaviours, and symptom and expectation outcomes following a critical incident and Critical Incident Stress Debriefing (CISD). | Two groups of 30 Australian police officers from the New South Wales Police Service who had been involved in shooting incidents were examined. One group received CISD and the other did not. | The group that received CISD showed a significant reduction in anger levels and greater use of some specific adaptive coping strategies. | The results suggest that CISD may have had some impact as a crisis intervention strategy for reducing the effects of long-term stress symptoms. However, a direct test of its effectiveness was not possible because of the multitude of external factors that were a significant part of officers’ lives. |
| McCraty, R (1999) | USA | This study explored the impact on a group of police officers from Santa Clara County, California of HeartMath® stress and emotional self-management training, which provides practical techniques designed to reduce stress in the moment, improve physiological and emotional balance, increase mental clarity and enhance performance and quality of life. | HeartMath® stress and emotional self-management training. Areas assessed included: physical health and vitality, emotional wellbeing, coping and interpersonal skills, work performance, workplace effectiveness and climate, family relationships, and physiological and psychological recalibration following acute stress. In addition, physiological measurements were obtained to determine the real-time cardiovascular impact of acutely stressful situations encountered in simulated police calls used in police training, and to identify officers at increased risk of cardiovascular disease and premature mortality. | Results showed that the HeartMath training improved officers’ capacity to recognize and manage their stress and negative emotions in both work and personal contexts. Over the course of the study, officers trained in the stress management techniques experienced reductions in stress, negative emotions and physical stress symptoms, as well as increased positive emotion and physical vitality as compared to a control group that did not receive the training. | This study provides evidence that practical stress and emotional self-management techniques can reduce damaging physiological and psychological responses to both acute and chronic stress in police, and positively impact a variety of major life areas in a relatively short period of time. |
| Richmond, RL (1999) | Australia | To evaluate the effects of a brief intervention to reduce excessive drinking, smoking and stress among police. | 1) Controlled intervention trial with pre- and post-intervention assessment approximately 8 months apart; (2) focus group identification of relevant factors. Assessment was carried out of 954 NSW (New South Wales, Australia) police at 19 stations within two matched districts in the Sydney metropolitan area. Five focus groups were carried out with 43 randomly selected police from the matched districts. Weekly alcohol consumption and binge drinking, smoking and symptoms of stress were measured by a self-administered Health and Fitness Questionnaire. | Alcohol consumption, particularly among men, was high at both baseline and follow-up assessments, although comparisons between groups across occasions showed no significant intervention effects. Excessive drinkers and those reporting moderate to severe stress levels reported more sick leave days (p<0.05, p<0.05). A significant increase in awareness of alcohol policies in the work-place showed in both experimental and control groups over time (p<0.01). The percentage of smokers declined significantly in both intervention and control groups. Overall, women had significantly more symptoms of stress than men. | The brief interventions did not produce significant improvements in three lifestyle factors beyond positive trends in alcohol consumption among women and general reductions in smoking among both study groups. Combining quantitative and qualitative approaches helped identify interactive individual and organizational factors which influence behavioural and cultural norms. |
| Tolin, DF (1999) | USA | The present single subject report describes successful implementation of exposure therapy with a police officer diagnosed with chronic PTSD following a work-related incident. | Exposure therapy; an intensive time-series design was used. Treatment consisted of a three-session measurement-only period to examine severity and stability of symptoms, followed by a five-session treatment phase, and a second three-session measurement-only phase to assess the stability of therapeutic change. The officer completed self-report inventories of symptom severity at each assessment point. More comprehensive assessments were administered at pre-treatment, immediately following treatment, and 6 months post-treatment. | The authors found that symptom relief was clearly associated with the onset of exposure therapy, and persisted after termination of this therapy through a 6-month follow-up period. | This case is a detailed demonstration of the cognitive behavioral treatment of PTSD in an emergency service worker, suggesting that this form of treatment holds promise for clinicians treating such personnel. Future studies should use group designs in which exposure therapy is compared to wait-list control or nondirective counseling, as has been done with other populations. |
| Welch, J (1998) | South Africa | Members of the HR and the employee support services travelled the length and breadth of the Free State running life skills and stress management workshops, and training commanders to recognize warning details and implement prevention techniques. | Suicide prevention – includes: awareness training (unclear duration) delivered by members of human resources and/or employee support services; gatekeeper training (unclear duration) delivered by members of human resources and/or employee support services; 24hr crisis telephone hotline; life skills and stress management workshops (unclear duration) delivered by members of human resources and/or employee support services; crisis intervention team; suicide post-intervention services; changes to media reporting guidelines following the suicide of an officer. | From the start of year conducted to the end of May, three officers committed suicide, compared to 13 in the same period the year before. The team expects the death toll to continue to fall. It is believed that 400 suicides were prevented by the campaign. | Some of the service's wider problems are also starting to be addressed as a result of the suicide prevention research. Management buy-in to the project has grown stronger. |
| Ford, N (1996) | Australia | This paper reports the use of carbemazepine and sodium valproate in a man with PTSD whose symptoms had failed to respond to two tricyclic antidepressants. | In the third week of admission, carbemazepine was added to the other medications. The dose of carbemazepine was increased to 400 mg/day giving steady state levels of 27 µmoles/L. He was readmitted to the hospital, and sodium valproate was commenced and increased to 500 mg twice daily, achieving steady-state levels of 520 µmoles/L. | Three days after commencing carbemazepine dose, he began to become more relaxed with an improvement in sleep and mood, a decrease in nightmares, and a diminishing startle response. He started to spend time out of the ward, and at one point took trial leave with his family. He was withdrawn from carbemazepine due to side effects; improved on sodium valproate. Following sodium valproate treatment, two days after reaching this dose his edginess began to decrease and he began to leave his room. Over the next 2 weeks his mood and sleep improved. The nightmares ended, and although intrusive recollections continued they were less frequent and distressing. He was discharged and followed up as an outpatient. | In treating this man, there was a clear association between the commencement of anticonvulsants and marked improvement in symptoms, particularly those of startle response, irritability, sleep disturbance, and depression. Following cessation of carbamazepine, his symptoms returned, leading to discharge on a cumbersome drug regime. The introduction of sodium valproate was associated with improvement. Sedation was not complained of with either drug, arguing against this as an acute effect producing improvement. Improvement was seen within 2 days of reaching this dose. It is possible on each occasion that this man's PTSD symptoms improved secondary to improvement in his depression. |
| Digliani, JA (1994) | USA | Fifty-one Colorado patrol and detention officers from the Fort Collins Police Services. Loveland Police Department and Larimer County Sheriff’s Department participated in a stress inoculation training program. | Officers were stratified by agency and primary function, then randomly assigned to either the treatment or delayed control group. Treatment group officers participated in a five-session, two-hour per session stress inoculation training program conducted over approximately seven weeks. Measures of trait anxiety, trait anger, current stressors, overall stress level, and self-efficacy were obtained for both groups prior to, following, and approximately four weeks after stress inoculation training. | Analyses of covariance demonstrated significant main effects on the first of the current stressor measures, and significant interaction effects on the second current stressor measure. Overall stress level indicated change in the predicted direction; however the degree of change lacked statistical significance. There were no significant differences between the treatment and control groups on trait anxiety, trait anger, and self-efficacy. | Treatment group feedback revealed that officers found the training program beneficial. The results generally supported stress inoculation training as an effective stress management approach for law enforcement officers. |
| Holbrook, MI (1994) | USA | This study aimed to  increase subjects' knowledge of sleep hygiene as measured by the Sleep Hygiene Awareness and Practice Scale by introducing instruction on stimulus-control techniques  and information regarding the effects of stimulants and hypnotics on sleep and sleep maintenance. | 31 male and 7 female law-enforcement officers voluntarily participated in a training session on sleep hygiene practices. On the Sleep Hygiene Awareness and Practice Scale administered prior to and after training, there were significant increases in awareness of sleep hygiene and knowledge of nicotine, caffeine, and hypnotics. We predicted that use of this knowledge would increase sleep satisfaction. | A comparison of scores on sleep hygiene indicated a significant increase in pre- to post-test awareness of sleep hygiene (t = 9.23, p<.001) and in knowledge about nicotine (t = 4.24, p<.001), hypnotics (t = 4.64, p< .001), and caffeine (t = 7.53, p < .001). However, 1-mo. follow-up scores on the Post-sleep Inventory reflected no change. It appears that scheduling demands, coupled with feelings of low self-efficacy toward managing those demands, resulted in little or no practice of sleep hygiene. | A more productive approach may be to incorporate a comprehensive behavioral program within departments to instill and reinforce better practice of sleep hygiene. |
| Norvell, N (1993) | USA | The effects of circuit weight training on mood, perceived stress, job satisfaction, and physical symptoms were investigated in a sample of state law enforcement officers. | Forty-three male officers who were not regularly exercising were assigned to either 4 months of circuit weight training or a wait-list control condition. | Four months of circuit weight training led to a significant increase in strength on cardiovascular fitness. Subjects also demonstrated significant improvements in mood, including decreases in somatization, anxiety, depression, and hostility. Circuit weight training also resulted in a decrease in reports of physical symptoms and in improvements in job satisfaction. | Results indicated that subjects who dropped out of the exercise training program evidenced significantly greater anxiety, depression, and hostility at pretreatment than subjects who completed the program. These findings suggest that circuit weight training programs may contribute to important psychological benefits. |
| Bohl, N (1991) | USA | The long-term effectiveness of brief psychological interventions in police officers who have been involved in critical incidents was assessed. | Three months after the critical incident, two groups that were similar with respect to age, number of years worked, and number of prior incidents were compared: Officers who had been treated within 24 hours after the incident and officers who had not been treated at all. | On formal, written tests, the treated group was significantly less depressed (p < .001) and angry (p < .02) than the untreated group. Also, the treated group reported significantly fewer stress-related symptoms (p < .001) than the untreated group. The two groups did not differ significantly on a measure of anxiety. | Overall, the data provided evidence for the effectiveness of brief interventions in police officers. It is suggested that treatment programs be mandatory for all officers involved in critical incidents. |
| Norris, R (1990) | UK | To determine whether fitness alters psychological and physiological indices of well-being, male police officers were assigned to either an aerobic or anaerobic training condition or to a no treatment control group. | The training groups met three times per week in 45 min sessions aimed at improving either cardiovascular endurance or muscle strength. Aerobic fitness level. Heart rate, blood pressure and self-report of stress and well-being were measured prior to and following 10 weeks of training. | Post-training fitness measures confirmed the effectiveness of training and between group differences for physiological and self-report measures were found. Subjects undergoing aerobic training evinced larger changes on the self-report measures of well-being and stress than the anaerobic trainers and both groups showed significant improvement when compared to controls. | This experiment provides support for the hypothesis that exercise, and in particular aerobic exercise, has positive effects of well-being. It is suggested that future research might usefully explore the particular contribution of different aspects of the training situation to these effects. |
| Coulson, JE (1987) | USA | A group of veteran police officers were studied in relation to the effectiveness of a stress reduction program which utilized a cognitive-behavioral approach to training. The Coulson Police Job Stress Discussionaire (CPJSD), a new instrument used to assess stressors, was also field tested. | A veteran group of police officers who received stress reduction training was compared to two control groups on pre-test and post-test Profile of Mood States (POMS) mood disturbance scores. The control groups were a veteran group who received no stress reduction training, and an academy group which received standard basic training but not the stress reduction program. | Contrary to the main hypothesis formulated, there were no significant differences found between the three groups on post-test POMS measures of mood disturbance when compared with pre-test measures. The construct validity of the POMS for use with police officers is challenged. | The specific format utilized is discussed and suggestions are offered for future study design. Specific difficulties inherent in the study of police groups are examined. The usefulness of the CPJSD for police job stress reduction program is suggested, as is the need for further field testing of this instrument. |
| Ackerley, DG (1986) | USA | This study was designed to address whether the sample, police personnel in a Midwestern suburban police force, are under high levels of stress, and whether the administration of a stress management program would effect changes in ways of coping with stress, quality of family life as it relates to work, job satisfaction, perceived control over events in their lives, and level of professional burnout. | The independent variable was a six-week stress management program. The dependent variables were assessed using the Social Readjustment Rating Scale, the Maslach Burnout Inventory, Police Officer's Questionnaire, the Rotter I-E Scale and eight behavioral indicators of job performance. A randomised block design was used to assign subjects, 49 police personnel within four intact squads, as a group to one of two conditions: a treatment group and control group. | Analyses of variances on hypotheses related to the three aspects of the burnout syndrome were not found to be significant. Analyses of variances on hypotheses related to a change in job satisfaction as a result of treatment were not found to be significant. Analysis of covariance on hypotheses related to a change in attitude toward family life as a result of treatment were not found to be significant. Individual item analysis of variance on the frequency of use of coping activities showed significant differences for smoking behavior. Analysis of variance on the locus of control measure showed no significant changes as a result of treatment. | From the findings in this study, it may be concluded that the hypotheses related to burnout, quality of family life as it relates to work, job satisfaction, perceived control over events were not supported. Of the coping activities, only smoking behavior showed an increase as a result of treatment. |
| Short, MA (1984) | USA | The purpose of the study was to investigate possible psychological changes in obese men after participating in an eight-week nutrition and physical conditioning program. | The subjects, 45 male, metropolitan policemen who were considered at least 20 percent over their optimum body weights, were placed on diets and received weekly instruction on topics of nutrition and exercise. The subjects were randomly divided into two groups, one that participated in aerobic conditioning and one that did not. The amount of oxygen consumption, as an index of physical fitness, and performance on selected subscales of the Tennessee Self-concept Scale (TSCS), were measured before and after the training and conditioning programs. | Both groups displayed significant increases in oxygen consumption and on the Physical Self and Self-satisfaction subscales, but on all these measures, the Conditioning Group increased significantly (2 to 3 times) more. For both groups, the Total Variability measure from the TSCS showed significant reductions, which have been associated with personality integration. | These results demonstrate that physical conditioning and dietary educational sessions or educational sessions alone are associated with positive changes in self-concept in obese individuals and also corroborate other studies that show links between physical and psychological fitness. |
| Sarason, IG  (1979) | USA | Police Academy trainees participated in a stress management program which focused on developing skills for coping with anxiety and anger. | Stress management training took place in six 2-hour sessions and included instruction and practice in the self-monitoring of reactions to stressful situations, muscular relaxation, and the development of adaptive self-statements. Self-report measures of anxiety and anger were obtained before and after the stress management program. In addition, self and observer ratings of trainees' performance in stressful simulated police activities were utilized as post-treatment dependent measures. | In comparison to a control group of trainees, the performance of the treatment group was rated, by academy personnel, as superior in several of the simulated police activities. | The results of the present study suggest that stress management with law enforcement officers may be most effective when the program focuses on the specific situations which are likely to be encountered by trainees. Limitations of the present program are examined and suggestions for future efforts with law enforcement personnel are discussed. |

**Abbreviations**: ASI – anxiety sensitivity index; BAI – Beck Anxiety Inventory; EMDR – eye movement desensitization and reprocessing; M – mean; PTSD – post-traumatic stress disorder; SD – standard deviation; TRiM – Trauma Risk Management Program

**Notes**: ^†^This study was an unpublished thesis, therefore the information presented here was taken from review level data.

## Table E2. Firefighter studies

| **Study author (year)** | **Study country** | **Study objective(s)** | **Methods** | **Results** | **Conclusion** |
| --- | --- | --- | --- | --- | --- |
| Sullivan, JP (2016)^38^ | USA | Study tested the hypothesis that a workplace-based Sleep Health Program (SHP) incorporating sleep health education and sleep disorders screening would improve firefighter health and safety compared to standard practice. | Prospective station-level randomised, field-based intervention in a US fire department with 1189 firefighters. Interventions included sleep health education, questionnaire-based sleep disorders screening, and sleep clinic referrals for respondents who screened positive for a sleep disorder. Firefighters were randomised by station. | Using departmental records, in an intention-to-treat analysis, firefighters assigned to intervention stations which participated in education sessions and had the opportunity to complete sleep disorders screening reported 46% fewer disability days than those assigned to control stations (1.4 ± 5.9 vs. 2.6 ± 8.5 days/firefighter, respectively; p = 0.003). There were no significant differences in departmental injury or motor vehicle crash rates between the groups. In post hoc analysis accounting for intervention exposure, firefighters who attended education sessions were 24% less likely to file at least one injury report during the study than those who did not attend, regardless of randomization (OR [95% CI] 0.76 [0.60, 0.98]; χ2 = 4.56; p = 0.033). There were no significant changes pre- versus post-study in self-reported sleep or sleepiness in those who participated in the intervention. | A firefighter workplace-based SHP providing sleep health education and sleep disorders screening opportunity can reduce injuries and work loss due to disability in firefighters. |
| Finney, EJ (2015)^54^ | USA | This article captures the history of suicide within the Houston Fire Department and summarizes each phase of the HFD Suicide Prevention Program in its development, purpose, methodology, and implementation. | The purpose of Phase I was to gather input and ideas from active duty firefighters on suicide in the fire service and to learn what they would like to see from the department regarding mental health issues. Via a special bulletin, all stations were ordered by the Fire Chief to attend a suicide prevention presentation. The purpose of Phase 2 was to educate firefighters about suicide and bring about more awareness of the subject to the entire department. Phase 2 of the Suicide Prevention Program was done face to face (in person) as a presentation to all of the stations and districts. The purpose of Phase 3 was to educate officers about how to identify the difference between “problems” and “crises”, to advise officers on how to deal with crisis situations, to describe proper documentation of mental health events, and to help officers identify and utilize mental health resources. Phase 3 of the Suicide Prevention Program was done as an online course. Via a special bulletin, all officers were made aware of Phase 3 and were ordered by the Fire Chief to complete this course. | The Suicide Prevention Program was successful in educating a large majority of the HFD firefighters about the history of suicide in the department, how to recognize suicide risks among firefighters, and the mental health resources available to members. In addition, it resulted in the creation of a more coordinated team (FSN) intended to address the mental health needs of HFD members. After the HFD Suicide Prevention Program began in 2007, there were no suicides of active duty members for five years, and at least three virtually certain suicides were averted. | While we cannot say for certain that this is the direct result of the program, it is clear that there has been a much greater emphasis on preventing the tragedy of suicide within the department since the program was put in place. We plan to maintain an awareness of the problem of suicide among our firefighters through continuing educational efforts and by outreach efforts by the department psychologist and the Firefighter Support Network. |
| Tuckey, MR (2014)^82^ | Australia | We conducted the first randomised controlled trial of critical incident stress debriefing (CISD) with emergency workers (67 volunteer fire-fighters) following shared exposure to an occupational potentially traumatic event (PTE). The goals of group CISD are to prevent post-traumatic stress and promote return to normal functioning following a PTE. | To assess both goals, the study measured four outcomes, before and after the intervention: post-traumatic stress, psychological distress, quality of life, and alcohol use. Fire brigades were randomly assigned to one of three treatment conditions: (1) CISD, (2) Screening (i.e., no-treatment), or (3) stress management Education. | Controlling for pre-intervention scores, CISD was associated with significantly less alcohol use post-intervention relative to Screening, and significantly greater post-intervention quality of life relative to Education. There were no significant effects on post-traumatic stress or psychological distress. | Overall, CISD may benefit broader functioning following exposure to work-related PTEs. Future research should focus on individual, group, and organizational factors and processes that can promote recovery from operational stressors. Ultimately, an occupational health (rather than victim-based) approach will provide the best framework for understanding and combating potential threats to the health and well-being of workers at high risk for PTE exposure. |
| Coupland, NJ (2009)^93^ | Canada | The purpose of this study is twofold: to determine whether a specified stress reduction model is effective and to provide information on stress reduction for those who are involved in stress-reduction programs. | Prazosin is prescribed at a dose of 1 mg at bedtime for 1 week, increasing in 1 mg increments every 3-4 days thereafter. | It initially makes patient feel light-headed and tired on rising; but this passes. Dry mouth is tolerable after eating breakfast. By week 3, patient reports improved sleep and continues taking 6 mg at bedtime after week 4. At follow-up, patient reports being less restless and having deeper, longer and more refreshing sleep, with some weeks free of traumatic nightmares. worries less about sleep and only moves to a separate bed on "bad nights." | Blockade of central (a1-adrenergic receptors with antagonists such as prazosin is better supported, with 3 pilot placebo-controlled augmentation trials (2 crossover, 1 parallel group design) showing superiority for prazosin at bedtime for the targeted symptoms of insomnia and nightmares. Prazosin also improved total and rapid eye movement (REM) sleep duration. The use of prazosin to treat PTSD is off-label, but labelled prescribing information stresses avoidance of hypotension by using a 1 mg first dose, slow titration and caution with concomitant medications with hypotensive effects and sedatives/hypnotics. |
| Kitchiner, NJ (2004)^96^ | UK | This paper will describe 3 cases which all suffered with PTSD and were treated via the partnership with a controversial therapy, eye movement desensitisation and reprocessing (EMDR). | EMDR for 3 patients, with 3-month follow-up. | All 3 patients who underwent EMDR reported a decline in intrusive symptoms and memories, fewer nightmares, less physiological arousal, and improved sleep and interpersonal interactions. These were sustained over the course of their sessions and until the 3-month follow-up period. | The case examples above all benefited from a relatively short time in treatment and were able to return to work (if off due to trauma) or work with less distress. EMDR has been an emerging therapy which would appear to be a potentially useful psychological treatment for PTSD, despite its lack of credible scientific explanation on how it actually works in reducing symptoms. |
| Harris, MB (2002)^81^ | USA | This study examined the relationships between debriefings and several mental health variables in a large sample of firefighters. | Of the 1,747 firefighters who were surveyed in the Federal Emergency Management Agency (FEMA) study, 852 met the selection criteria of (a) completing all measures and (b) reporting experience of a stressful incident in the course of their work during the previous 6 months that both the participant and peers agreed was a critical incident. We tested for differences on all scales and demographic categories between the sample and the original 1,749 firefighters, as well as for differences in demographics between the debriefed and non-debriefed groups within the 660 cases in the final sample. To test for differences between the groups on Trauma Exposure and Debriefing of Firefighters, ethnicity, gender, volunteer status, education, marital status, language, and age group, the chi-square test was used. | Debriefing had a weak inverse correlation with negative affectivity and a weak positive correlation with positive world assumptions. No relationship was found between debriefing and PTSD. | Although causal conclusions could not be taken from the results of the present study, the findings emphasize the need for further research on CISD, stress exposure, and dispositional variables in firefighters. Future research should address individual differences and the hypothesis of diathesis-stress. |
| Regehr, C (2001)^83^ | NR | This study attempts to investigate the efficacy of crisis debriefing (CD) group interventions in a sample of firefighters following a critical incident. | This study addresses the efficacy of CD groups for 164 Australian firefighters following a critical incident. | Findings indicated that the majority of firefighters attending crisis debriefing groups perceived that the intervention was beneficial in reducing their level of stress. However, there was no significant association between attending CD groups and scores on the Beck Depression Inventory. Attending a CD group was associated with higher scores on intrusion subscale of the Impact of Events Scale. | This study, as others, has determined that firefighters attending crisis debriefing groups feel subjectively better. However, they do not appear to experience reduced levels of post-traumatic symptoms and depression when compared to their colleagues who did not attend. This suggests that no singular, simplistic approach to managing the aftermath of traumatic events can meet the needs of all affected emergency service personnel. |
| Bohl, N (1995)^80^ | USA | Objective tests were used to assess symptoms and firefighters treated with CISD were compared with untreated firefighters. | Such a program provides that individuals who have been involved in a traumatic incident meet with a psychologist for a counseling session some time during the first 48 hours after the incident. Individuals are seen only once, typically for one or two hours, but the session is intense. Participants are asked to describe the traumatic episode and encouraged to vent the strong feelings aroused by the incident. They are reassured about the normality of those feelings, provided with information about the possible occurrence of delayed symptoms, and helped to assimilate the occurrence so it can be seen in the context of prior experiences. In the study reported here, a formal evaluation was done. Objective tests were used to assess symptoms, and treated firefighters were compared with untreated firefighters. | The results showed that a brief intervention, 1 1/2 hours in length, given 24 hours after a critical incident reduces delayed stress symptoms in firefighters. On all four measures tested – depression, anger, anxiety, and long-term stress symptoms – the untreated group scored significantly higher, which meant that men in the untreated group had more signs of delayed stress than the treated group. Those untreated were more anxious, depressed, and angry and were experiencing more nightmares, flashbacks, and changes in sleeping and eating habits. | It is recommended, therefore, that treatment be mandatory for firefighters who have been involved in a critical incident. The findings presented here provide clear evidence that, as is the case with police officers, such treatment works. A mandatory program would take the burden of decision making out of the individual's hands. |

**Abbreviations**: CISD – critical incident stress debriefing; EMDR – eye movement desensitization and reprocessing; PTSD – post-traumatic stress disorder

## Table E3. Correctional officer studies

| **Study author (year)** | **Study country** | **Study objective(s)** | **Methods** | **Results** | **Conclusion** |
| --- | --- | --- | --- | --- | --- |
| Ruck, S. (2013) | UK | This study was designed to evaluate the effectiveness and a program of support for prison service staff following a traumatic incident within a prison environment. | Prison staff who had been exposed to a range of traumatic events were offered debriefing. Measurements were taken soon after the incident and again one month later. The scores of those receiving debriefing were compared with those who did not receive debriefing. | Prison staff receiving debriefing showed a significant reduction in their traumatic stress, anxiety and depression scores. There was no significant difference in the symptoms of the non-debriefed group. | The findings suggest that group-based well-structured debrief sessions can be useful in reducing symptoms of post-traumatic stress following exposure to critical incidents in the workplace. The results suggest that there are benefits in undertaking group debriefing within an organisational setting. |
| McCraty, R (2003) | USA | This study investigated the impact of HeartMath’s Power to Change Performance stress and health risk reduction program on physiological and psychological stress and health risk factors in a sample of correctional peace officers. | Eighty-eight officers from three facilities were randomised to an experimental group and a wait-list control group. The experimental group participated in the stress and health risk reduction program, which was delivered over two consecutive days. The program included instruction on health risk factors as well as training in positive emotion-focused stress reduction techniques intended to reduce negative emotional arousal, improve physiological balance, increase positive affect, and enhance performance. Measures of physiological and psychological stress and health risk were assessed before the program and again 3 months afterward. Three self-report psychological surveys were also included to assess emotional stress and work-related variables. | An analysis of baseline data revealed that officers in this study were under greater stress and at greater health risk in comparison to a reference sample of working adults. A within-group analysis of pre-post changes showed that 3 months after the intervention program, employees in the experimental group demonstrated significant reductions in stress and health risk factors, as well as significant improvements in work-related parameters. There were also improvements in key organizationally relevant measures in the experimental group after the program, including significant increases in productivity, motivation, goal clarity, and perceived manager support. Finally, the reduction in health risk factors achieved in this study are projected to lead to reductions in both health care and absentee costs, yielding a total projected annual cost savings of USD$699 per employee. | The results of this study indicate that the Power to Change Performance program was effective in significantly reducing stress and health risk factors in a population of correctional peace officers, while enhancing employee productivity and psychological well-being. These changes were realized with minimal intervention and in a relatively brief period of time, and should result in significant cost savings to the organization if the program is expanded to larger employee populations. |

# Appendix F: List of excluded primary studies

1. Physical health status of World Trade Center rescue and recovery workers and volunteers - New York City, July 2002-August 2004. MMWR Morb Mortal Wkly Rep. 2004;53(35):807-12.
2. Amster ED, Fertig SS, Baharal U, Linn S, Green MS, Lencovsky Z, et al. Occupational exposures and symptoms among firefighters and police during the carmel forest fire: the Carmel cohort study. Isr Med Assoc J. 2013;15(6):288-92.
3. Arboleda A, Morrow PC, Crum MR, Shelley MC, 2nd. Management practices as antecedents of safety culture within the trucking industry: similarities and differences by hierarchical level. J Safety Res. 2003;34(2):189-97.
4. Arora VM, Georgitis E, Woodruff JN, Humphrey HJ, Meltzer D. Improving sleep hygiene of medical interns: can the sleep, alertness, and fatigue education in residency program help? Arch Intern Med. 2007;167(16):1738-44.
5. Assistance BoJ. In Harm’s Way: Law Enforcement Suicide Prevention. http://policesuicide.spcollege.edu/crisisIHW.htm (11 November 2013, date last accessed). 2013.
6. Atlantis E, Chow CM, Kirby A, Singh MF. An effective exercise-based intervention for improving mental health and quality of life measures: a randomized controlled trial. Prev Med. 2004;39(2):424-34.
7. Atlantis E, Chow C-M, Kirby A, fiatarone singh M. Worksite intervention effects on sleep quality: A randomized controlled trial. Journal of occupational health psychology. 2006;11:291-304.
8. Bell L, Virden T, Lewis D, Cassidy B. Effects of 13-Hour 20-Minute Work Shifts on Law Enforcement Officers' Sleep, Cognitive Abilities, Health, Quality of Life, and Work Performance: The Phoenix Study. Police Quarterly. 2015;18.
9. Bender A, Eynan R, O'Grady J, Nisenbaum R, Shah R, Links PS. Best practice intervention for post-traumatic stress disorder among transit workers. Work. 2016;54(1):59-71.
10. Benedek DM, Ritchie EC. "Just-in-time" mental health training and surveillance for the Project HOPE mission. Mil Med. 2006;171(10 Suppl 1):63-5.
11. Bisson JI, Jenkins PL, Alexander J, Bannister C. Randomised controlled trial of psychological debriefing for victims of acute burn trauma. Br J Psychiatry. 1997;171:78-81.
12. Blonk RWB, Brenninkmeijer V, Lagerveld SE, Houtman ILD. Return to work: A comparison of two cognitive behavioural interventions in cases of work-related psychological complaints among the self-employed. Work & Stress. 2006;20(2):129-44.
13. Bond FW, Bunce D. Mediators of change in emotion-focused and problem-focused worksite stress management interventions. J Occup Health Psychol. 2000;5(1):156-63.
14. Boyce R WT, Mullins A, Jones G, Cottrell R. . Health Promotion Strategies Derived from a Metropolitan Police Weight Loss Competition with Weight Loss Comparisons by Gender and BMI Category. The Internet Journal of Allied Health Sciences and Practice. 2014;12(3):Article 10.
15. Boyce RW, Dyer EA, Willett TK, Figueroa JL, Jones GR. Police weight-loss competition in the United States with gender, age, and job comparisons with health promotion recommendations. International Journal of Health Promotion and Education. 2015;53(4):204-15.
16. Brouwers EP, Tiemens BG, Terluin B, Verhaak PF. Effectiveness of an intervention to reduce sickness absence in patients with emotional distress or minor mental disorders: a randomized controlled effectiveness trial. Gen Hosp Psychiatry. 2006;28(3):223-9.
17. Brown MJ, Tandy RD, Wulf G, Young JC. The effect of acute exercise on pistol shooting performance of police officers. Motor Control. 2013;17(3):273-82.
18. Butterworth S, Linden A, McClay W, Leo MC. Effect of motivational interviewing-based health coaching on employees' physical and mental health status. J Occup Health Psychol. 2006;11(4):358-65.
19. Buyukcakir C. Hearing loss in Turkish aviators. Mil Med. 2005;170(7):572-6.
20. Byczek L, Walton SM, Conrad KM, Reichelt PA, Samo DG. Cardiovascular risks in firefighters: implications for occupational health nurse practice. Aaohn j. 2004;52(2):66-76.
21. Capodaglio EM, Imbriani M, Criffo A. A method for the assessment of fitness in aerobically taxing occupations. Int J Occup Med Environ Health. 1996;9(3):227-34.
22. Carter PA, Dyer KA, Mikan SQ. Sleep disturbance, chronic stress, and depression in hospice nurses: testing the feasibility of an intervention. Oncol Nurs Forum. 2013;40(5):E368-73.
23. Center RT. Post Traumatic Stress Management/Psychological First Aid Workshop (PTSM/ PFA) & Recognizing Depression and Suicide Risk 2013 [Available from: http://www.riversidetraumacenter.org/.
24. Chen PH, Kuo HY, Chueh KH. Sleep hygiene education: efficacy on sleep quality in working women. J Nurs Res. 2010;18(4):283-9.
25. Chopko B, Palmieri P, Adams R. Associations Between Police Stress and Alcohol Use: Implications for Practice. Journal of Loss and Trauma. 2013;18.
26. Chopko BA, Palmieri PA, Facemire VC. Prevalence and Predictors of Suicidal Ideation Among U.S. Law Enforcement Officers. Journal of Police and Criminal Psychology. 2014;29(1):1-9.
27. Cocke C, Dawes J, Orr RM. The Use of 2 Conditioning Programs and the Fitness Characteristics of Police Academy Cadets. J Athl Train. 2016;51(11):887-96.
28. Colin L, Nieuwenhuys A, Visser A, Oudejans RRD. Positive Effects of Imagery on Police Officers' Shooting Performance under Threat. Applied Cognitive Psychology. 2014;28(1):115-21.
29. Cone JE, Li J, Kornblith E, Gocheva V, Stellman SD, Shaikh A, et al. Chronic probable PTSD in police responders in the world trade center health registry ten to eleven years after 9/11. Am J Ind Med. 2015;58(5):483-93.
30. Conlon L, Fahy TJ, Conroy R. PTSD in ambulant RTA victims: a randomized controlled trial of debriefing. J Psychosom Res. 1999;46(1):37-44.
31. Crawley AA, Sherman RA, Crawley WR, Cosio-Lima LM. Physical Fitness of Police Academy Cadets: Baseline Characteristics and Changes During a 16-Week Academy. J Strength Cond Res. 2016;30(5):1416-24.
32. Deahl M, Srinivasan M, Jones N, Thomas J, Neblett C, Jolly A. Preventing psychological trauma in soldiers: the role of operational stress training and psychological debriefing. Br J Med Psychol. 2000;73 ( Pt 1):77-85.
33. Dedic G, Panic M. Suicide prevention program in the Army of Serbia and Montenegro. Mil Med. 2007;172(5):551-5.
34. Demling RH, DeSanti L. Effect of a hypocaloric diet, increased protein intake and resistance training on lean mass gains and fat mass loss in overweight police officers. Ann Nutr Metab. 2000;44(1):21-9.
35. Difede J, Cukor J, Jayasinghe N, Patt I, Jedel S, Spielman L, et al. Virtual reality exposure therapy for the treatment of posttraumatic stress disorder following September 11, 2001. J Clin Psychiatry. 2007;68(11):1639-47.
36. Difede J, Malta LS, Best S, Henn-Haase C, Metzler T, Bryant R, et al. A randomized controlled clinical treatment trial for World Trade Center attack-related PTSD in disaster workers. J Nerv Ment Dis. 2007;195(10):861-5.
37. Duckworth DH. Psychological problems arising from disaster work. Stress Medicine. 1986;2(4):315-23.
38. Dunleavy K, Taylor A, Gow J, Cullen B, Roy K. Police officer anxiety after occupational blood and body fluid exposure. Occup Med (Lond). 2012;62(5):382-4.
39. Dyregrov A, Gjestad R. A maritime disaster: reactions and follow-up. Int J Emerg Ment Health. 2003;5(1):3-14.
40. Eick AA, Wang Z, Hughes H, Ford SM, Tobler SK. Comparison of the trivalent live attenuated vs. inactivated influenza vaccines among U.S. military service members. Vaccine. 2009;27(27):3568-75.
41. Eid J, Johnsen BH, Weisaeth L. The effects of group psychological debriefing on acute stress reactions following a traffic accident: a quasi-experimental approach. Int J Emerg Ment Health. 2001;3(3):145-54.
42. Elliott JL, Lal S. Blood Pressure, Sleep Quality and Fatigue in Shift Working Police Officers: Effects of a Twelve Hour Roster System on Cardiovascular and Sleep Health. Int J Environ Res Public Health. 2016;13(2):172.
43. Erneston AG, Ricks MR, Tate TJ, Ana RS. Vision readiness in the United States Air Force revisited. Mil Med. 1996;161(1):27-8.
44. Fabreau G, Elliott M, Khanna S, Minty E, Wallace JE, de Grood J, et al. Shifting perceptions: a pre-post study to assess the impact of a senior resident rotation bundle. BMC Med Educ. 2013;13:115.
45. Fertout M, Jones N, Greenberg N, Mulligan K, Knight T, Wessely S. A review of United Kingdom Armed Forces' approaches to prevent post-deployment mental health problems. Int Rev Psychiatry. 2011;23(2):135-43.
46. Feuerstein M, Nicholas RA, Huang GD, Dimberg L, Ali D, Rogers H. Job stress management and ergonomic intervention for work-related upper extremity symptoms. Appl Ergon. 2004;35(6):565-74.
47. Force AD. Australian Defence Force Suicide Prevention Program (ADF SPP). Accessed 25 July, 2015 from http://www.defence.gov.au/Health/DMH/SuicidePreventionProgram.asp. 2015.
48. Forceville G. Suicide Prevention in the Workplace: Good Practices in Pre- and Post-Intervention (Flanders). https://webgate.ec.europa.eu/sanco_mental_health/public/POLICY/1426/show.html. 2013.
49. Fox J, Desai MM, Britten K, Lucas G, Luneau R, Rosenthal MS. Mental-health conditions, barriers to care, and productivity loss among officers in an urban police department. Conn Med. 2012;76(9):525-31.
50. Frappell-Cooke W, Gulina M, Green K, Hacker Hughes J, Greenberg N. Does trauma risk management reduce psychological distress in deployed troops? Occup Med (Lond). 2010;60(8):645-50.
51. Gamble RP, Boreham CA, Stevens AB. Effects of a 10-week exercise intervention programme on exercise and work capacities in Belfast's ambulance-men. Occup Med (Lond). 1993;43(2):85-9.
52. Gardner B, Rose J, Mason O, Tyler P, Cushway D. Cognitive therapy and behavioural coping in the management of work-related stress: An intervention study. Work & Stress. 2005;19(2):137-52.
53. Gerkin D. Firefighters: fitness for duty. Occup Med. 1995;10(4):871-6.
54. Gersons B, Nijdam, M., Meewisse, M., & Olff, M. Protocol: Brief eclectic psycho- therapy for posttraumatic stress disorder (BEPP). Academic Medical Centre Unpublished Manual2010.
55. Godard C, Chevalier A, Lecrubier Y, Lahon G. APRAND programme: an intervention to prevent relapses of anxiety and depressive disorders. First results of a medical health promotion intervention in a population of employees. Eur Psychiatry. 2006;21(7):451-9.
56. Gould M, Greenberg N, Hetherton J. Stigma and the military: evaluation of a PTSD psychoeducational program. J Trauma Stress. 2007;20(4):505-15.
57. Greenberg N, Dow C, Bland D. Psychological risk assessment following the terrorist attacks in New York in 2001. Journal of Mental Health. 2009;18:216-23.
58. Greenberg N, Henderson A, Langston V, Iversen A, Wessely S. Peer responses to perceived stress in the Royal Navy. Occup Med (Lond). 2007;57(6):424-9.
59. Greenberg N, Langston V, Everitt B, Iversen A, Fear NT, Jones N, et al. A cluster randomized controlled trial to determine the efficacy of Trauma Risk Management (TRiM) in a military population. J Trauma Stress. 2010;23(4):430-6.
60. Greenberg N, Langston V, Iversen AC, Wessely S. The acceptability of 'Trauma Risk Management' within the UK Armed Forces. Occup Med (Lond). 2011;61(3):184-9.
61. Greven F, Kerstjens HA, Duijm F, Eppinga P, de Meer G, Heederik D. Respiratory effects in the aftermath of a major fire in a chemical waste depot. Scand J Work Environ Health. 2009;35(5):368-75.
62. Grime PR. Computerized cognitive behavioural therapy at work: a randomized controlled trial in employees with recent stress-related absenteeism. Occup Med (Lond). 2004;54(5):353-9.
63. Grunert BK, Devine CA, McCallum-Burke S, Matloub HS, Sanger JR, Yousif NJ. On-site work evaluations: desensitisation for avoidance reactions following severe hand injuries. J Hand Surg Br. 1989;14(2):239-41.
64. Grunert BK, Devine CA, Smith CJ, Matloub HS, Sanger JR, Yousif NJ. Graded work exposure to promote work return after severe hand trauma: a replicated study. Ann Plast Surg. 1992;29(6):532-6.
65. Grunert BK, Matloub HS, Sanger JR, Yousif NJ. Treatment of posttraumatic stress disorder after work-related hand trauma. J Hand Surg Am. 1990;15(3):511-5.
66. Grunert BK, Weis JM, Smucker MR, Christianson HF. Imagery rescripting and reprocessing therapy after failed prolonged exposure for post-traumatic stress disorder following industrial injury. J Behav Ther Exp Psychiatry. 2007;38(4):317-28.
67. Gullestrup J, Lequertier B, Martin G. MATES in construction: impact of a multimodal, community-based program for suicide prevention in the construction industry. Int J Environ Res Public Health. 2011;8(11):4180-96.
68. Guppy A, Marsden J. Assisting employees with drinking problems: Changes in mental health, job perceptions and work performance. Work & Stress. 1997;11(4):341-50.
69. Hardaway CA, Gregory KB. Fatigue and Sleep Debt in an Operational Navy Squadron. The International Journal of Aviation Psychology. 2005;15(2):157-71.
70. Harrell JS, Johnston LF, Griggs TR, Schaefer P, Carr EG, Jr., McMurray RG, et al. An occupation based physical activity intervention program: improving fitness and decreasing obesity. Aaohn j. 1996;44(8):377-84.
71. Hatch BC, Hilber DJ, Elledge JB, Stout JW, Lee RB. The effects of visual acuity on target discrimination and shooting performance. Optom Vis Sci. 2009;86(12):E1359-67.
72. Health TNCfRaRM. Farm-Link Suicide Prevention Project. 2013.
73. Hobbs M, Mayou R, Harrison B, Worlock P. A randomised controlled trial of psychological debriefing for victims of road traffic accidents. BMJ. 1996;313(7070):1438-9.
74. Hogberg G, Pagani M, Sundin O, Soares J, Aberg-Wistedt A, Tarnell B, et al. Treatment of post-traumatic stress disorder with eye movement desensitization and reprocessing: outcome is stable in 35-month follow-up. Psychiatry Res. 2008;159(1-2):101-8.
75. Hourani LL, Council CL, Hubal RC, Strange LB. Approaches to the primary prevention of posttraumatic stress disorder in the military: a review of the stress control literature. Mil Med. 2011;176(7):721-30.
76. Humphries C, Carr A. The short term effectiveness of critical incident stress debriefing. The Irish Journal of Psychology. 2012;22:188-97.
77. Incolink. Incolink: A Joint Enterprise of Employer Associations and Unions in the Building and Construction Industry 2013 [Available from: http://www.incolink.org.au/Home.aspx.
78. James LC, Kowalski TJ. Suicide prevention in an army infantry division: a multi-disciplinary program. Mil Med. 1996;161(2):97-101.
79. Jones D, Kennedy, KR., Hourani, L., Hawkes, C., Long, MA., & Robbins, D. Suicide Prevention in the Navy and Marine Corps: Applying the Public Health Model. Navy medicine. 2001;November-December:31-6.
80. Kang M. Effectiveness of Motivational Interviewing on Changes in Fitness, Blood Lipids, and Exercise Adherence of Police Officers: An Outcome-Based Action Study. Journal of Correctional Health Care. 2008;14:48-62.
81. Kawakami N, Araki S, Kawashima M, Masumoto T, Hayashi T. Effects of work-related stress reduction on depressive symptoms among Japanese blue-collar workers. Scand J Work Environ Health. 1997;23(1):54-9.
82. Kawakami N, Haratani T, Iwata N, Imanaka Y, Murata K, Araki S. Effects of mailed advice on stress reduction among employees in Japan: a randomized controlled trial. Ind Health. 1999;37(2):237-42.
83. Keenan P, Royle L. Vicarious trauma and first responders: a case study utilizing eye movement desensitization and reprocessing (EMDR) as the primary treatment modality. Int J Emerg Ment Health. 2007;9(4):291-8.
84. Kelly KJ, Connelly E, Reinhold GA, Byrne M, Prezant DJ. Assessment of health effects in New York City firefighters after exposure to polychlorinated biphenyls (PCBs) and polychlorinated dibenzofurans (PCDFs): the Staten Island Transformer Fire Health Surveillance Project. Arch Environ Health. 2002;57(4):282-93.
85. Kitchener BA, Jorm AF. Mental health first aid training in a workplace setting: A randomized controlled trial [ISRCTN13249129]. BMC Psychiatry. 2004;4(1):23.
86. Knapik JJ, Rieger W, Palkoska F, Van Camp S, Darakjy S. United States Army physical readiness training: rationale and evaluation of the physical training doctrine. J Strength Cond Res. 2009;23(4):1353-62.
87. Knox KL, Litts DA, Talcott GW, Feig JC, Caine ED. Risk of suicide and related adverse outcomes after exposure to a suicide prevention programme in the US Air Force: cohort study. BMJ. 2003;327(7428):1376.
88. Knox KL, Pflanz S, Talcott GW, Campise RL, Lavigne JE, Bajorska A, et al. The US Air Force suicide prevention program: implications for public health policy. Am J Public Health. 2010;100(12):2457-63.
89. Kraemer WJ, Vescovi JD, Volek JS, Nindl BC, Newton RU, Patton JF, et al. Effects of concurrent resistance and aerobic training on load-bearing performance and the Army physical fitness test. Mil Med. 2004;169(12):994-9.
90. Kuehl KS, Elliot DL, Goldberg L, MacKinnon DP, Vila BJ, Smith J, et al. The safety and health improvement: enhancing law enforcement departments study: feasibility and findings. Front Public Health. 2014;2:38-.
91. Lammers-van der Holst HM, Kerkhof GA. Shift work tolerance and the importance of sleep quality: a study of police officers. Biological Rhythm Research. 2015;46(2):257-64.
92. Landman A, Nieuwenhuys A, Oudejans RR. Decision-related action orientation predicts police officers' shooting performance under pressure. Anxiety Stress Coping. 2016;29(5):570-9.
93. Lang T, Nicaud V, Slama K, Hirsch A, Imbernon E, Goldberg M, et al. Smoking cessation at the workplace. Results of a randomised controlled intervention study. Worksite physicians from the AIREL group. J Epidemiol Community Health. 2000;54(5):349-54.
94. Lapenaite D VR. Lowering suicide risk: Situation and prevention measures in the Lithuanian Armed Forces. In: Wiederhold B, editor. Lowering suicide risk in returning troops: Wounds of war. Amsterdam, Netherlands: IOS Press; 2008.
95. Lavender T, Walkinshaw SA. Can midwives reduce postpartum psychological morbidity? A randomized trial. Birth. 1998;25(4):215-9.
96. Lavoie-Tremblay M, Bourbonnais R, Viens C, Vezina M, Durand PJ, Rochette L. Improving the psychosocial work environment. J Adv Nurs. 2005;49(6):655-64.
97. Lee C, Slade P, Lygo V. The influence of psychological debriefing on emotional adaptation in women following early miscarriage: a preliminary study. Br J Med Psychol. 1996;69 ( Pt 1):47-58.
98. Lee KA, Gay CL, Alsten CR. Home-based behavioral sleep training for shift workers: a pilot study. Behav Sleep Med. 2014;12(6):455-68.
99. Lilly MM, Pole N, Best SR, Metzler T, Marmar CR. Gender and PTSD: What can we learn from female police officers? J Anxiety Disord. 2009;23(6):767-74.
100. LivingWorks. Applied Suicide Intervention Skills Training (ASIST) 2013 [Available from: http://www.livingworks.net/.
101. Louhevaara V, Soukainen J, Lusa S, Tulppo M, Tuomi P, Kajaste T. Development and evaluation of a test drill for assessing physical work capacity of fire-fighters. International Journal of Industrial Ergonomics. 1994;13(2):139-46.
102. Ltd. OF. OzHelp Foundation 2013 [Available from: https:// ozhelp.org.au/site/.
103. Macklin M, Metzger L, Lasko N, Berry N, Orr S, Pitman R. Five-year follow-up study of eye movement desensitization and reprocessing therapy for combat-related posttraumatic stress disorder. Comprehensive psychiatry. 2000;41:24-7.
104. Maguen S, Metzler TJ, McCaslin SE, Inslicht SS, Henn-Haase C, Neylan TC, et al. Routine work environment stress and PTSD symptoms in police officers. J Nerv Ment Dis. 2009;197(10):754-60.
105. Maia DB, Marmar CR, Henn-Haase C, Nobrega A, Fiszman A, Marques-Portella C, et al. Predictors of PTSD symptoms in brazilian police officers: the synergy of negative affect and peritraumatic dissociation. Braz J Psychiatry. 2011;33(4):362-6.
106. Maia DB, Nobrega A, Marques-Portella C, Mendlowicz MV, Volchan E, Coutinho ES, et al. Peritraumatic tonic immobility is associated with PTSD symptom severity in Brazilian police officers: a prospective study. Braz J Psychiatry. 2015;37(1):49-54.
107. Mainsbridge CP, Cooley PD, Fraser SP, Pedersen SJ. The effect of an e-health intervention designed to reduce prolonged occupational sitting on mean arterial pressure. J Occup Environ Med. 2014;56(11):1189-94.
108. Matthews LR. Effect of Staff Debriefing on Posttraumatic Stress Symptoms After Assaults by Community Housing Residents. Psychiatric Services. 1998;49(2):207-12.
109. Mayou RA, Ehlers A, Hobbs M. Psychological debriefing for road traffic accident victims. Three-year follow-up of a randomised controlled trial. Br J Psychiatry. 2000;176:589-93.
110. McDiarmid MA, Engelhardt SM, Oliver M, Gucer P, Wilson PD, Kane R, et al. Health surveillance of Gulf War I veterans exposed to depleted uranium: updating the cohort. Health Phys. 2007;93(1):60-73.
111. Mehlum L. Forebygging av selvmord blant unge—Nyere erfaringer fra Forsvaret [Prevention of suicide in young people— Recent experiences from the armed forces]. Tidsskr Nor Loegeforen. 1998;118(1724-1726).
112. Menard K, Arter M. Stress, Coping, Alcohol Use, and Posttraumatic Stress Disorder Among an International Sample of Police Officers: Does Gender Matter? Police Quarterly. 2014;17:307-27.
113. Mino Y, Babazono A, Tsuda T, Yasuda N. Can stress management at the workplace prevent depression? A randomized controlled trial. Psychother Psychosom. 2006;75(3):177-82.
114. Mulick PS, Naugle AE. Behavioral activation for comorbid PTSD and major depression: A case study. Cognitive and Behavioral Practice. 2004;11(4):378-87.
115. Mulligan K, Fear NT, Jones N, Wessely S, Greenberg N. Psycho-educational interventions designed to prevent deployment-related psychological ill-health in Armed Forces personnel: a review. Psychol Med. 2011;41(4):673-86.
116. Mumford EA, Taylor BG, Kubu B. Law enforcement officer safety and wellness. Police Quarterly. 2015;18(2):111-33.
117. Mutrie N, Carney C, Blamey A, Crawford F, Aitchison T, Whitelaw A. "Walk in to Work Out": a randomised controlled trial of a self help intervention to promote active commuting. J Epidemiol Community Health. 2002;56(6):407-12.
118. Naghii MR. The importance of body weight and weight management for military personnel. Mil Med. 2006;171(6):550-5.
119. Nakao M, Nishikitani M, Shima S, Yano E. A 2-year cohort study on the impact of an Employee Assistance Programme (EAP) on depression and suicidal thoughts in male Japanese workers. Int Arch Occup Environ Health. 2007;81(2):151-7.
120. Ng SM, Chan TH, Chan CL, Lee AM, Yau JK, Chan CH, et al. Group debriefing for people with chronic diseases during the SARS pandemic: Strength-Focused and Meaning-Oriented Approach for Resilience and Transformation (SMART). Community Ment Health J. 2006;42(1):53-63.
121. Nieuwenhuys A, Cañal-Bruland R, Oudejans RRD. Effects of Threat on Police Officers' Shooting Behavior: Anxiety, Action Specificity, and Affective Influences on Perception. Applied Cognitive Psychology. 2012;26(4):608-15.
122. Nieuwenhuys A, Oudejans R. Effects of anxiety on handgun shooting behavior of police officers: a pilot study. Anxiety Stress Coping. 2010;23(2):225-33.
123. Nieuwenhuys A, Oudejans RR. Training with anxiety: short- and long-term effects on police officers' shooting behavior under pressure. Cogn Process. 2011;12(3):277-88.
124. Nieuwenhuys A, Savelsbergh GJ, Oudejans RR. Persistence of threat-induced errors in police officers' shooting decisions. Appl Ergon. 2015;48:263-72.
125. Noordik E, van der Klink JJ, Klingen EF, Nieuwenhuijsen K, van Dijk FJ. Exposure-in-vivo containing interventions to improve work functioning of workers with anxiety disorder: a systematic review. BMC Public Health. 2010;10:598.
126. Nurmi LA. The sinking of the Estonia: the effects of critical incident stress debriefing (CISD) on rescuers. Int J Emerg Ment Health. 1999;1(1):23-31.
127. Orr RM, Ford K, Stierli M. Implementation of an Ability-Based Training Program in Police Force Recruits. J Strength Cond Res. 2016;30(10):2781-7.
128. Pedersen SJ, Cooley PD, Mainsbridge C. An e-health intervention designed to increase workday energy expenditure by reducing prolonged occupational sitting habits. Work. 2014;49(2):289-95.
129. Peters KK, Carlson JG. Worksite stress management with high-risk maintenance workers: A controlled study. International Journal of Stress Management. 1999;6(1):21-44.
130. Petterson IL, Donnersvärd HÅ, Lagerström M, Toomingas A. Evaluation of an intervention programme based on empowerment for eldercare nursing staff. Work & Stress. 2006;20(4):353-69.
131. Pietrzak RH, Schechter CB, Bromet EJ, Katz CL, Reissman DB, Ozbay F, et al. The burden of full and subsyndromal posttraumatic stress disorder among police involved in the World Trade Center rescue and recovery effort. J Psychiatr Res. 2012;46(7):835-42.
132. Plat MJ, Frings-Dresen MH, Sluiter JK. Reproducibility and validity of the stair-climb test for fire fighters. Int Arch Occup Environ Health. 2010;83(7):725-31.
133. Plat MJ, Frings-Dresen MH, Sluiter JK. Clinimetric quality of the fire fighting simulation test as part of the Dutch fire fighters Workers' Health Surveillance. BMC Health Serv Res. 2010;10:32.
134. Poulsen AA, Sharpley CF, Baumann KC, Henderson J, Poulsen MG. Evaluation of the effect of a 1-day interventional workshop on recovery from job stress for radiation therapists and oncology nurses: A randomised trial. J Med Imaging Radiat Oncol. 2015;59(4):491-8.
135. Priest SR, Henderson J, Evans SF, Hagan R. Stress debriefing after childbirth: a randomised controlled trial. Med J Aust. 2003;178(11):542-5.
136. Punakallio A. Balance abilities of different-aged workers in physically demanding jobs. J Occup Rehabil. 2003;13(1):33-43.
137. Radun I, Ohisalo J, Radun J, Kecklund G. Night work, fatigued driving and traffic law: the case of police officers. Ind Health. 2011;49(3):389-92.
138. Rajaratnam SM, Barger LK, Lockley SW, Shea SA, Wang W, Landrigan CP, et al. Sleep disorders, health, and safety in police officers. Jama. 2011;306(23):2567-78.
139. Renden PG, Landman A, Daalder NR, de Cock HP, Savelsbergh GJP, Oudejans RRD. Effects of threat, trait anxiety and state anxiety on police officers’ actions during an arrest. Legal and Criminological Psychology. 2017;22(1):116-29.
140. Renden PG, Landman A, Geerts SF, Jansen SE, Faber GS, Savelsbergh GJ, et al. Effects of anxiety on the execution of police arrest and self-defense skills. Anxiety Stress Coping. 2014;27(1):100-12.
141. Renden PG, Nieuwenhuys A, Savelsbergh GJ, Oudejans RR. Dutch police officers' preparation and performance of their arrest and self-defence skills: a questionnaire study. Appl Ergon. 2015;49:8-17.
142. Richards DA, Rose JS. Exposure therapy for post-traumatic stress disorder. Four case studies. Br J Psychiatry. 1991;158:836-40.
143. Richmond VL, Rayson MP, Wilkinson DM, Carter JM, Blacker SD, Nevill A, et al. Development of an operational fitness test for the Royal Air Force. Ergonomics. 2008;51(6):935-46.
144. Rose S, Brewin CR, Andrews B, Kirk M. A randomized controlled trial of individual psychological debriefing for victims of violent crime. Psychol Med. 1999;29(4):793-9.
145. Rosekind MR, Gregory KB, Mallis MM. Alertness management in aviation operations: enhancing performance and sleep. Aviat Space Environ Med. 2006;77(12):1256-65.
146. Rossomanno CI, Herrick JE, Kirk SM, Kirk EP. A 6-month supervised employer-based minimal exercise program for police officers improves fitness. J Strength Cond Res. 2012;26(9):2338-44.
147. Rozanov VA, Mokhovikov AN, Stiliha R. Successful model of suicide prevention in the Ukraine military environment. Crisis. 2002;23(4):171-7.
148. Rumyantseva GM, Stepanov AL. Post-traumatic stress disorder in different types of stress (clinical features and treatment). Neurosci Behav Physiol. 2008;38(1):55-61.
149. Scott LD, Hofmeister N, Rogness N, Rogers AE. An interventional approach for patient and nurse safety: a fatigue countermeasures feasibility study. Nurs Res. 2010;59(4):250-8.
150. Selkirk R, McLaren S, Ollerenshaw A, McLachlan AJ, Moten J. The longitudinal effects of midwife‐led postnatal debriefing on the psychological health of mothers. Journal of Reproductive and Infant Psychology. 2006;24(2):133-47.
151. Senjo S, Dhungana K. A Field Data Examination of Policy Constructs Related to Fatigue Conditions in Law Enforcement Personnel. Police Quarterly. 2009;12:123-36.
152. Senjo SR. Dangerous fatigue conditions: a study of police work and law enforcement administration. Police Practice and Research. 2011;12(3):235-52.
153. Seok J-M, Cho J-H, Jeon W-J, Ahn J-O. Risk factors for fatigue and stress among Korean police officers. J Phys Ther Sci. 2015;27(5):1401-5.
154. Shalev AY, Peri T, Rogel-Fuchs Y, Ursano RJ, Marlowe D. Historical group debriefing after combat exposure. Mil Med. 1998;163(7):494-8.
155. Sharpley JG, Fear NT, Greenberg N, Jones M, Wessely S. Pre-deployment stress briefing: does it have an effect? Occup Med (Lond). 2008;58(1):30-4.
156. Sheppard WD, Staggers FJ, John L. The effects of a stress management program in a high security government agency. Anxiety, Stress, & Coping. 1997;10(4):341-50.
157. Sijbrandij M, Olff M, Reitsma JB, Carlier IV, Gersons BP. Emotional or educational debriefing after psychological trauma. Randomised controlled trial. Br J Psychiatry. 2006;189:150-5.
158. Small R, Lumley J, Donohue L, Potter A, Waldenström U. Randomised controlled trial of midwife led debriefing to reduce maternal depression after operative childbirth. BMJ. 2000;321(7268):1043-7.
159. Smith KC, Wallace DP. Improving the sleep of children’s hospital employees through an email-based sleep wellness program. Clinical Practice in Pediatric Psychology. 2016;4(3):291-305.
160. Smith-Coggins R, Rosekind MR, Buccino KR, Dinges DF, Moser RP. Rotating shiftwork schedules: can we enhance physician adaptation to night shifts? Acad Emerg Med. 1997;4(10):951-61.
161. Sofer S, Eliraz A, Kaplan S, Voet H, Fink G, Kima T, et al. Greater weight loss and hormonal changes after 6 months diet with carbohydrates eaten mostly at dinner. Obesity (Silver Spring). 2011;19(10):2006-14.
162. Soteriades ES, Hauser R, Kawachi I, Liarokapis D, Christiani DC, Kales SN. Obesity and cardiovascular disease risk factors in firefighters: a prospective cohort study. Obes Res. 2005;13(10):1756-63.
163. Sothmann MS, Gebhardt DL, Baker TA, Kastello GM, Sheppard VA. Performance requirements of physically strenuous occupations: validating minimum standards for muscular strength and endurance. Ergonomics. 2004;47(8):864-75.
164. Spates CR, Burnette MM. Eye movement desensitization: three unusual cases. J Behav Ther Exp Psychiatry. 1995;26(1):51-5.
165. Spencer-Thomas S. Working Minds: Suicide Prevention in the Workplace 2013 [Available from: http://workingminds.org.
166. Sperlich B, Fricke H, de Marees M, Linville JW, Mester J. Does respiratory muscle training increase physical performance? Mil Med. 2009;174(9):977-82.
167. Staff OotDCo. Army G-1 Suicide Intervention Program 2013 [Available from: http://www.armyg1.army.mil/hr/suicide/default.asp.
168. Steffen MW, Hazelton AC, Moore WR, Jenkins SM, Clark MM, Hagen PT. Improving sleep: outcomes from a worksite healthy sleep program. J Occup Environ Med. 2015;57(1):1-5.
169. Strating M, Bakker RH, Dijkstra GJ, Lemmink KA, Groothoff JW. A job-related fitness test for the Dutch police. Occup Med (Lond). 2010;60(4):255-60.
170. Tehrani N. Compassion fatigue: experiences in occupational health, human resources, counselling and police. Occup Med (Lond). 2010;60(2):133-8.
171. van der Velden PG, Kleber RJ, Grievink L, Yzermans JC. Confrontations with aggression and mental health problems in police officers: The role of organizational stressors, life-events and previous mental health problems. Psychological Trauma: Theory, Research, Practice, and Policy. 2010;2(2):135-44.
172. Vanderburgh PM, Flanagan S. The backpack run test: a model for a fair and occupationally relevant military fitness test. Mil Med. 2000;165(5):418-21.
173. Vimercati L, Carrus A, Bisceglia L, Tato I, Bellotta MR, Russo A, et al. Biological monitoring and allergic sensitization in traffic police officers exposed to urban air pollution. Int J Immunopathol Pharmacol. 2006;19(4 Suppl):57-60.
174. Violanti JM, Fekedulegn D, Andrew ME, Charles LE, Hartley TA, Vila B, et al. Shift work and the incidence of injury among police officers. Am J Ind Med. 2012;55(3):217-27.
175. Waggoner LB, Grant DA, Van Dongen HPA, Belenky G, Vila B. A combined field and laboratory design for assessing the impact of night shift work on police officer operational performance. Sleep. 2012;35(11):1575-7.
176. Wang Z, Inslicht SS, Metzler TJ, Henn-Haase C, McCaslin SE, Tong H, et al. A prospective study of predictors of depression symptoms in police. Psychiatry Res. 2010;175(3):211-6.
177. Warner CH, Appenzeller GN, Parker JR, Warner C, Diebold CJ, Grieger T. Suicide prevention in a deployed military unit. Psychiatry. 2011;74(2):127-41.
178. Weaver JL, McAlister WH. Vision readiness of the reserve forces of the U.S. Army. Mil Med. 2001;166(1):64-6.
179. Wee DF, Mills DM, Koehler G. The effects of critical incident stress debriefing (CISD) on emergency medical services personnel following the Los Angeles Civil Disturbance. Int J Emerg Ment Health. 1999;1(1):33-7.
180. Weis JM, Grunert BK, Christianson HF. Early versus delayed imaginal exposure for the treatment of posttraumatic stress disorder following accidental upper extremity injury. Hand (N Y). 2012;7(2):127-33.
181. Whybrow D. Psychiatric nursing liaison in a combat zone: an autoethnography. J Psychiatr Ment Health Nurs. 2013;20(10):896-901.
182. Whybrow D, Jones N, Greenberg N. Corporate knowledge of psychiatric services available in a combat zone. Mil Med. 2013;178(2):e241-7.
183. Winwood PC, Tuckey MR, Peters R, Dollard MF. Identification and measurement of work-related psychological injury: piloting the psychological injury risk indicator among frontline police. J Occup Environ Med. 2009;51(9):1057-65.
184. Wolmer L, Hamiel D, Laor N. Preventing children's posttraumatic stress after disaster with teacher-based intervention: a controlled study. J Am Acad Child Adolesc Psychiatry. 2011;50(4):340-8, 8.e1-2.
185. Yadav A, Rani S, Singh S. Working "out-of-phase" with reference to chronotype compromises sleep quality in police officers. Chronobiol Int. 2016;33(2):151-60.
